# Asymmetric Cu─N─Ru Bridgedsite Nanozyme‐Loaded Injectable Thermogel Boosts Cuproptosis‐Like Death for Multidrug‐Resistant Urinary Tract Infections

**DOI:** 10.1002/advs.202520705

**Published:** 2026-01-28

**Authors:** Guanlin Li, Shike Zhang, Jinggong Liu, Xin Wang, Heng Wang, Hongcan Yang, Yuhao Zhou, Wen Liu, Hongxing Liu, Wenqi Wu

**Affiliations:** ^1^ Department of Urology the Second Affiliated Hospital of Guangzhou Medical University Guangzhou China; ^2^ State Key Laboratory of Traditional Chinese Medicine Syndrome Orthopedics Department The Second Affiliated Hospital of Guangzhou University of Chinese Medicine Guangzhou China; ^3^ Department of Urology Guangdong Provincial Key Laboratory of Urological Diseases Guangdong Engineering Research Center of Urinary Minimally Invasive Surgery The First Affiliated Hospital Robot and Intelligent Equipment Guangzhou Institute of Urology Guangzhou Medical University Guangzhou China; ^4^ School of Public Health Guangzhou Medical University Guangzhou China

**Keywords:** asymmetric site, cuproptosis, multidrug‐resistant, nanozyme, urinary tract infections

## Abstract

Multidrug‐resistant urinary tract infections (MDR‐UTIs) remain one of the most challenging clinical infections globally owing to the paucity of effective therapeutic agents. Nanozymes‐triggered cuproptosis‐like bacterial death has emerged as a promising bactericidal strategy. However, its efficacy is compromised by insufficient catalytic sites and suboptimal synergy among active centers. Consequently, the development of nanozymes capable of efficiently inducing cuproptosis‐like death holds considerable potential for treating MDR‐UTIs. Herein, the Cu‐ZIF8‐Ru nanozyme was engineered that leverages an asymmetric heterobimetallic Cu─N─Ru bridged catalytic center to achieve efficient multienzyme activities and trigger cuproptosis‐like death in MDR‐UTIs. Theoretical simulations revealed that introducing Ru sites as secondary catalytic centers upshifts the Cu d‐band center, thereby lowering activation barriers and enhancing catalytic activity at the Cu─N─Ru active site. Cu‐ZIF8‐Ru nanozyme demonstrated strong bactericidal effects against MDR‐bacteria by promoting intracellular Cu^2^^+^ accumulation and depleting cellular energy, ultimately leading to cuproptosis‐like death. A thermogel encapsulating Cu‐ZIF8‐Ru enabled intravesical in situ gelation and sustained release, providing durable bactericidal and anti‐inflammatory effects in both acute and recurrent MDR‐UTIs models with a favorable biosafety. In summary, Cu‐ZIF8‐Ru offers an efficient, controllable, and low‐toxicity intravesical bactericidal strategy that addresses key clinical demands in MDR‐UTIs management and holds substantial translational promise.

## Graphical abstract

To address multidrug‐resistant urinary tract infections (MDR‐UTIs), we developed Cu‐ZIF8‐Ru nanozyme featuring an asymmetric Cu─N─Ru catalytic site. This unique structure enhances multienzyme‐mimetic activity, eradicating resistant bacteria by inducing a cuproptosis‐like death pathway through intracellular Cu^2^^+^ accumulation and energy depletion. When encapsulated in a thermogel for sustained intravesical release, it demonstrates lasting antibacterial efficacy and biosafety, holding significant translational promise.

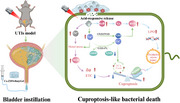

## 1 Introduction

Multidrug‐resistant urinary tract infections (MDR‐UTIs) have emerged as one of the most challenging common clinical infections worldwide, primarily due to the inappropriate use of broad‐spectrum antibiotics and the spread of resistance genes [1, 2, 3]. MDR‐UTIs frequently originate from residual bacteria and biofilms. Associated chronic inflammation not only diminishes treatment efficacy but also leads to recurrence following initial symptom resolution [4]. While intravenous carbapenems and β‐lactam inhibitor combinations retain efficacy against MDR bacteria, their high cost and limited availability restrict broad clinical application [5]. Furthermore, diagnostic delays and the slow susceptibility testing often postpone critical treatment, allowing recurrence to drive further resistance evolution and escalate healthcare burdens [6, 7, 8]. Therefore, developing effective therapeutic strategies against the clinical challenges of MDR‐UTIs is imperative. Copper‐dependent cell death (cuproptosis) has emerged as a distinct programmed cell‐death pathway initiated by disruption of copper homeostasis [9]. Mechanistically, intracellular Cu^2^^+^ overload preferentially targets lipoylated enzymes within the tricarboxylic acid (TCA) cycle, inducing protein aggregation, damaging iron‐sulfur (Fe─S) clusters, and impairing the respiratory electron‐transport chain. Consequently, these alterations suppress TCA‐cycle activity, cripple cellular bioenergetics, exacerbate oxidative stress, and ultimately precipitate cell death [10]. The TCA cycle functions not only in eukaryotic mitochondria but also in the cytoplasm of numerous prokaryotes. Therefore, leveraging Cu^2^^+^ overload to activate a cuproptosis‐like pathway presents a promising therapeutic avenue against MDR‐UTIs [11, 12, 13]. As excess copper is inherently cytotoxic, achieving targeted Cu^2^^+^ release at the infection site is critical for protecting healthy tissues. Given that MDR‐bacteria possess potent efflux pumps and robust oxidative‐stress defenses, cuproptosis‐like induction alone often yields suboptimal therapeutic outcomes [14]. Thus, the rational combination of copper‐mediated lethal mechanism with other antibacterial therapy is essential to harness the full potential of cuproptosis‐like pathway for treating MDR‐UTIs.

Nanozymes have been widely adopted in catalytic antibacterial therapy owing to the robust operational stability and high catalytic efficiency [15, 16, 17]. By mimicking oxidase‐like (OXD), peroxidase‐like (POD), and glutathione peroxidase‐like (GSH‐Px) activities, nanozymes generate reactive oxygen species (ROS) and deplete intracellular glutathione (GSH) [18, 19, 20]. This dual action disrupts the bacterial cell envelope and respiratory electron transport chain while inducing severe oxidative stress, collectively leading to bacterial death [21]. Functioning independently of conventional antibiotic targets, this approach evades major resistance mechanisms, including target‐site modifications, efflux pumps, and enzymatic degradation. Moreover, the continuous catalytic nature of nanozymes enables sustained bactericidal activity even at low doses [22]. Nanozymes also show strong potential for synergy with adjunct modalities like antimicrobial photodynamic therapy (aPDT), presenting a promising strategy against multidrug‐resistant infections [23, 24]. Although aPDT itself offers broad‐spectrum activity via ROS generation with a low resistance risk, its efficacy is often constrained by poor delivery selectivity and the complex microenvironments of biofilms and urine [25, 26, 27]. Concurrently, the limited number of active sites and the rapid decay of enzyme‐mimetic activity in many nanozymes frequently result in inadequate antibacterial performance in vitro and in vivo [28]. The recent emergence of precisely controllable, cuproptosis‐like bacterial death offers another avenue for multimodal therapy [29, 30]. Integrating it with nanozyme catalysis and aPDT may address the respective limitations of both modalities [31]. Thus, a multimodal antibacterial paradigm that merges cuproptosis‐like death with aPDT and catalytic therapy offers a robust solution for enhancing efficacy and mitigating resistance. However, most nanozymes possess only a single type of active site. This restricts their catalytic efficiency and impedes an optimal adsorption/desorption balance for reaction intermediates, ultimately limiting therapeutic potential [32, 33]. A promising strategy to overcome this involves precisely engineering the local electronic structure of metal active centers by increasing site density and introducing asymmetric coordination [34]. This approach is effectively realized in heterobimetallic nanostructures, which in turn facilitates the regulation of oxygenated intermediate adsorption/desorption and suppresses reaction activation barriers.

Metal–organic frameworks (MOFs) represent ideal platforms for constructing nanozymes with tailored multiple metal active centers due to their structural and compositional versatility [35]. The incorporation of adjacent heterobimetallic sites within a single MOF unit can optimize the local electronic structure and enhance catalytic performance [36]. Furthermore, ZIF‐8 has been widely investigated as a nanozyme carrier or precursor due to its high structural stability, biocompatibility, compositional tunability, and high loading capacity [37, 38]. Its pH‐responsive degradation profile is especially advantageous for applications in the mildly acidic urinary tract environment. Cu‐doped ZIF‐8 effectively mitigates the rapid protein‐mediated chelation of free Cu^2^^+^ under physiological conditions [39]. Notably, in eukaryotic cells, the ZnT1 transporter promotes intracellular Cu^2^^+^ accumulation and facilitates cuproptosis, whereas exogenous Zn^2^^+^ can competitively inhibit Cu^2^^+^ uptake [40]. Leveraging this distinction, Cu‐doped ZIF‐8 releases Zn^2^^+^ in an acidic urinary environment. The released Zn^2^^+^ competitively counters Cu^2^^+^ influx into adjacent healthy cells, thereby enhancing the biosafety profile of the treatment. Despite these advantages, the enzyme‐like activity of Cu‐doped ZIF‐8 is often constrained by low active‐site density and weak cooperativity between sites [41]. Moreover, overly large particles sediment in urine and are subject to rapid clearance via cyclical bladder emptying, drastically reducing their retention and efficacy at intravesical infection sites. Additionally, Ruthenium complexes are readily excited to highly oxidative excited state species and have the potential to act as synergistic metal‐ligand units to regulate the metal active sites of nanozymes [42, 43, 44]. Accordingly, incorporating ruthenium complexes to fine‐tune the local electronic distribution at distinct metal centers presents a promising avenue for significantly boosting nanozyme catalytic activity.

**SCHEME 1:**
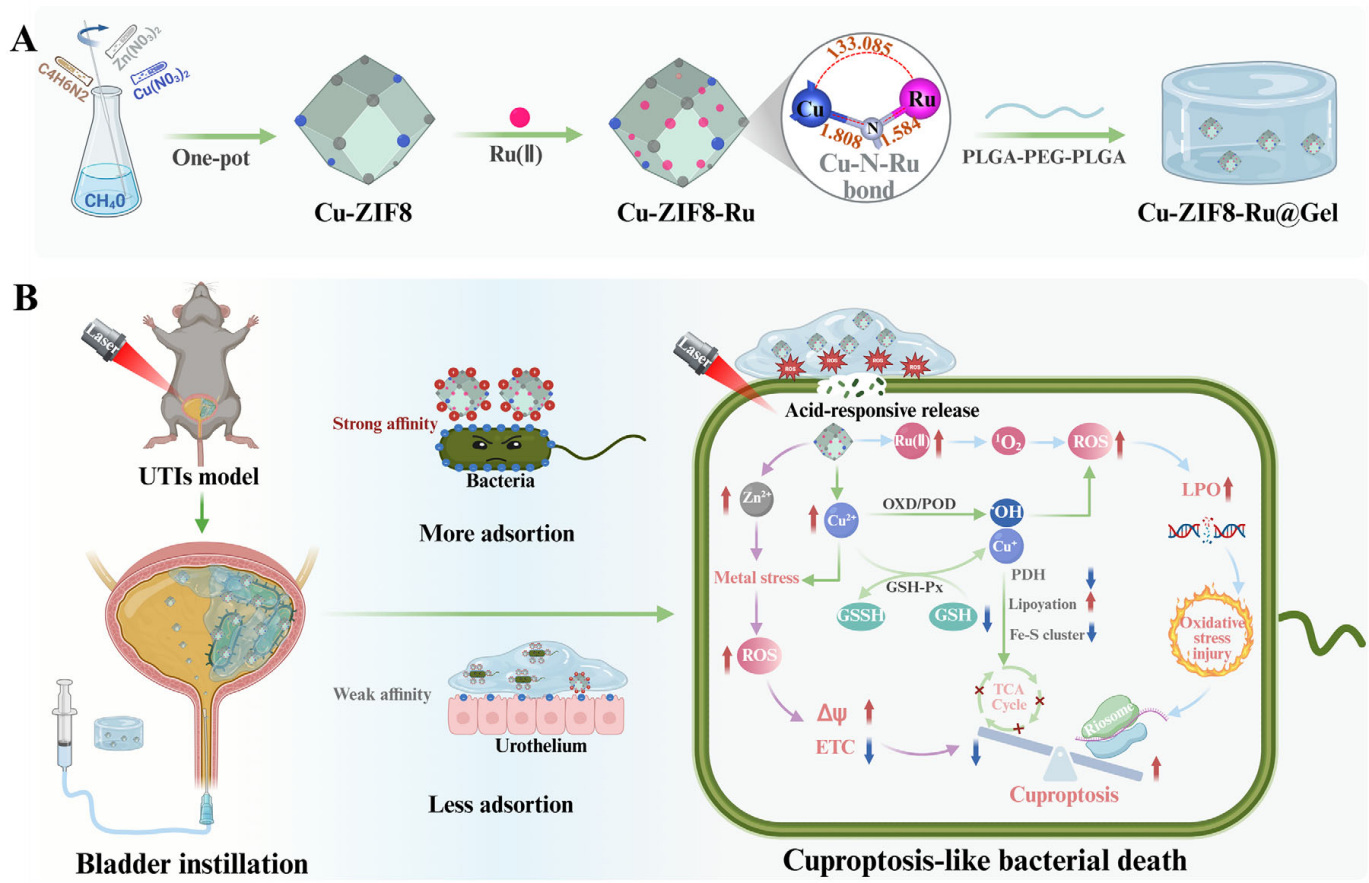
Synergistic Mechanism of Cu‐ZIF8‐Ru nanozyme enhancing cuproptosis‐like death for MDR‐UTIs therapy. (A) Fabrication of the Cu‐ZIF8‐Ru nanozyme and the Cu‐ZIF8‐Ru@Gel thermogel. (B) Mechanism of intravesical instillation of Cu‐ZIF8‐Ru@Gel achieves synergistic multienzyme activity to promote cuproptosis‐like antibacterial effects in MDR‐UTIs. (Created with BioRender.com).

Herein, the Cu‐ZIF8‐Ru nanozyme featuring an asymmetric heterobimetallic Cu─N─Ru active site was developed by integrating the ruthenium complex [Ru(phen)_2_dppz]^2^^+^ (Ru(II)) into the Cu‐ZIF‐8 precursor (Scheme 1A). X‐ray absorption fine structure (XAFS) and density functional theory (DFT) simulations confirmed that the Cu─N─Ru center acts as the catalytic core, wherein Ru incorporation expands the coordination space and introduces additional reaction sites in the vicinity of Cu. Notably, this configuration leads to an upshifted d‐band center, which promotes interfacial electron transfer and lowers reaction energy barriers, thereby synergistically enhancing OXD‐, POD‐, and GSH‐Px‐like activities. By adhering to multidrug‐resistant bacteria, the Cu‐ZIF8‐Ru nanozyme generates localized ROS that disrupts the cell envelope and increases membrane permeability, thereby facilitating Cu^2^^+^ influx and potentiating lethal oxidative stress. RNA‐seq analysis of treated MDR *E. coli* indicated that the Cu‐ZIF8‐Ru nanozyme induces upregulation of stress‐response and damage‐repair pathways alongside downregulation of nutrient transport and energy metabolism (Scheme 1B). The resulting energy metabolic disruption and intensified oxidative stress culminate in irreversible cell death, confirming the proposed cuproptosis‐like mechanism. In both acute and recurrent MDR‐UTI models, a thermogel loaded with the Cu‐ZIF8‐Ru nanozyme (Cu‐ZIF8‐Ru‐Gel) was administered intravesically. It underwent in situ gelation to enable sustained release, resulting in strong antibacterial and anti‐inflammatory effects with good biosafety. In summary, the Cu‐ZIF8‐Ru nanozyme presents an efficient, controllable, and low‐toxicity intravesical bactericidal strategy that addresses pressing clinical needs in MDR‐UTIs management and holds substantial translational promise.

## 2 Results and Discussion

### 2.1 Preparation and Characterization of Cu‐ZIF8‐Ru Nanozyme

**FIGURE 1:**
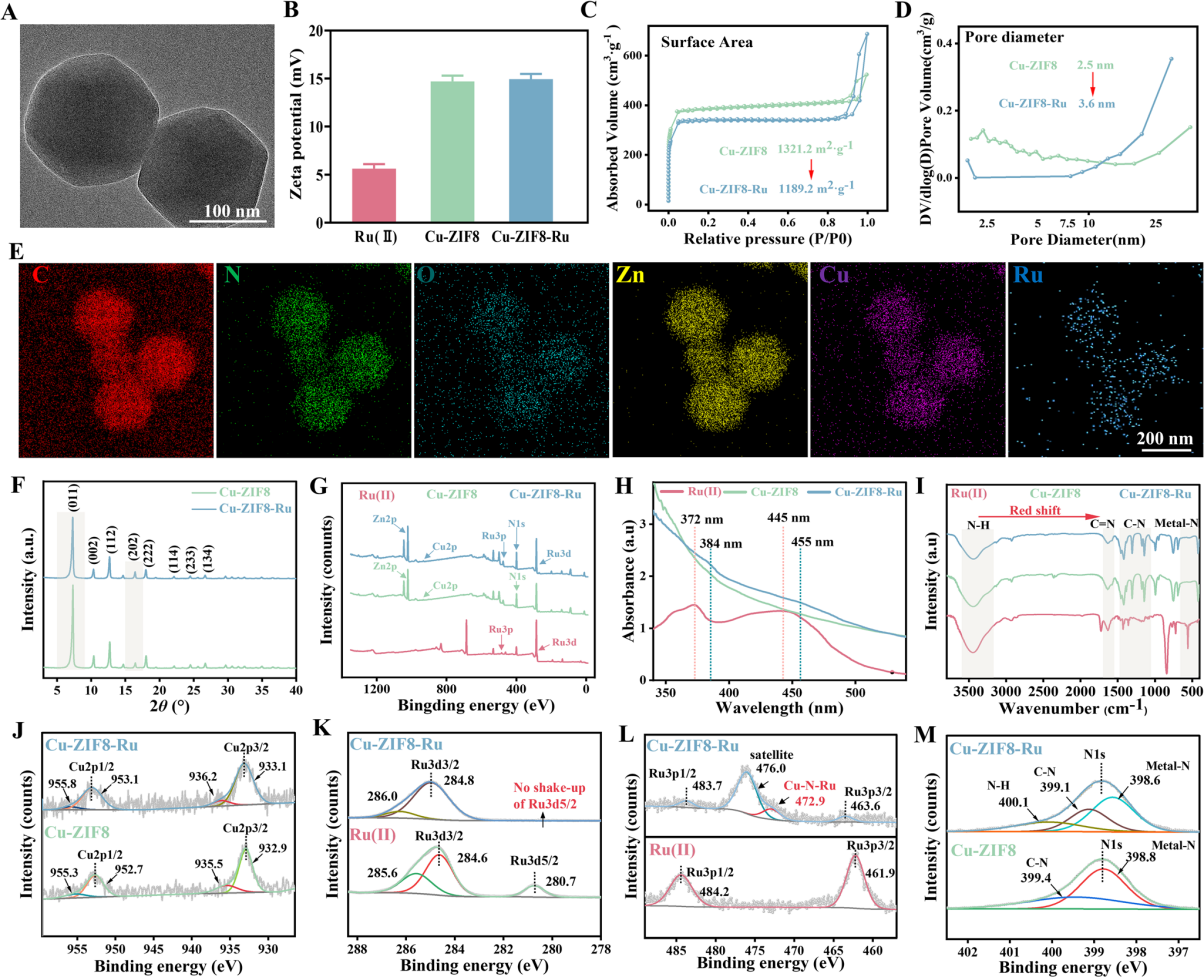
Characterization of Cu‐ZIF8‐Ru nanozyme. (A) TEM image of Cu‐ZIF8‐Ru. (B) Zeta potential of Ru(II), Cu‐ZIF8 and Cu‐ZIF8‐Ru. (C) N_2_ adsorption–desorption isotherms of Cu‐ZIF8 and Cu‐ZIF8‐Ru. (D) Pore size distribution of Cu‐ZIF8 and Cu‐ZIF8‐Ru. (E) EDS elemental mapping of C, N, O, Zn, Cu, and Ru in Cu‐ZIF8‐Ru. (F) XRD patterns of Cu‐ZIF8 and Cu‐ZIF8‐Ru. (G) XPS survey spectra of Ru(II), Cu‐ZIF8, and Cu‐ZIF8‐Ru. (H) UV–vis absorption spectra of Ru(II), Cu‐ZIF8, and Cu‐ZIF8‐Ru. (I) FTIR spectra of Ru(II), Cu‐ZIF8 and Cu‐ZIF8‐Ru. High‐resolution XPS spectra of (J) Cu 2p, (K) Ru 3d, (L) Ru 3p, and (M) N 1s.

Asymmetric metal coordination sites have attracted considerable interest due to their unique electronic structures and potential for high catalytic efficiency [45]. Herein, we designed and synthesized a Cu‐ZIF8‐Ru nanozyme featuring asymmetric heterobimetallic Cu─N─Ru bridged active sites, followed by comprehensive structural characterization. To assess the morphological impact of copper incorporation, we prepared Cu‐ZIF8 with different Cu loadings. TEM imaging (Figure S1) confirmed that the pristine rhombic dodecahedral morphology of ZIF‐8 was preserved in all samples, with an average particle size of ∼200 nm and no observable aggregation. This indicates that Cu^2^^+^ doping does not compromise the structural integrity of the ZIF‐8 framework. Ru(II) can act as a synergistic metal‐ligand unit to modulate the electronic structure of nanozymes. The Cu‐ZIF8‐Ru nanozyme was constructed by stirring a mixture of Cu‐ZIF8 and the Ru(II) in DMSO in the dark for over overnight. TEM images (Figure 1A) showed well‐dispersed, uniform polyhedra with smoother surfaces than Cu‐ZIF8 and no aggregates, confirming the formation of homogeneous Cu‐ZIF8‐Ru nanozyme formation. The ζ‐potential of Cu‐ZIF8‐Ru was significantly higher than that of Ru(II) alone and comparable to that of Cu‐ZIF8 (Figure 1B), reflecting good colloidal stability and dispersibility, in agreement with TEM observations. Hydrodynamic size analysis further recorded an average diameter of ∼209 nm for Cu‐ZIF8‐Ru (Figures S2 and S3), representing a slight increase of about 9 nm relative to Cu‐ZIF8. This modest size change is consistent with the surface adsorption of Ru(II) onto the Cu‐ZIF8 framework.

Brunauer–Emmett–Teller (BET) analysis was conducted to assess the structural changes of Cu‐ZIF8 upon Ru(II) incorporation. Both Cu‐ZIF8 and Cu‐ZIF8‐Ru exhibited type‐IV N_2_ adsorption/desorption isotherms with distinct H3 hysteresis loops (Figure 1C), indicative of slit‐shaped mesopores primarily formed by interparticle spaces, alongside a minor contribution from intrinsic micropores. Notably, the specific surface area of Cu‐ZIF8‐Ru (1189.2 m^2^·g^−^^1^) was slightly lower than that of Cu‐ZIF8 (1321.2 m^2^·g^−^^1^), indicating that Ru(II) occupies a portion of the high‐surface‐area sites without disrupting the framework. The substantial surface area of Cu‐ZIF8‐Ru ensures ample active site exposure and efficient substrate accessibility. Concurrently, the pore diameter increased from 2.5 nm for Cu‐ZIF8 to 3.6 nm for Cu‐ZIF8‐Ru (Figure 1D). This shift suggests that Ru(II) species are not aggregated within the pores but are preferentially anchored at the pore openings. This localization partially occupies micropores and subtly alters the pore entrance geometry, while preserving and even slightly expanding the mesoporous channels. Energy‐dispersive X‐ray spectroscopy (EDS) mapping (Figure 1E; Figure S4) revealed a uniform spatial distribution of all constituent elements (C, N, O, Zn, Cu, and Ru) in Cu‐ZIF8‐Ru, confirming the successful integration and homogeneous dispersion of Ru(II) without aggregation. Furthermore, the XRD pattern of Cu‐ZIF8‐Ru closely aligned with that of Cu‐ZIF8 (Figure 1F), confirming the preservation of the host crystal structure. A closer inspection revealed consistent, minor shifts of the (011) and (202) diffraction peaks toward lower angles (Figure S5). These shifts are indicative of a slight lattice expansion, providing direct evidence for the successful integration of Ru(II) into the framework and the associated subtle structural reorganization. These results confirm the uniform distribution of Ru(II) without surface aggregation, which is consistent with the BET and EDS analyses. Finally, the XPS survey spectrum of Cu‐ZIF8‐Ru (Figure 1G) clearly shows the presence of Cu 2p, Ru 3p, Ru 3d, N 1s, and Zn 2p core‐level signals, thereby providing the successful incorporation and coexistence of Ru(II) within the Cu‐ZIF8‐Ru framework.

The electronic structure and coordination environment of Cu‐ZIF8‐Ru were further probed by UV–vis and FT‐IR spectroscopy. Compared to the free Ru(II) (main band at ∼445 nm, shoulder at ∼372 nm), Cu‐ZIF8‐Ru exhibited a redshifted main band at 455 nm with a corresponding shoulder at 384 nm (Figure 1H). This bathochromic shift not only confirms the successful integration of Ru(II) but also points to a direct electronic interaction and a restructured local coordination environment. This pronounced redshift provides direct evidence of electronic coupling between the metals, signaling restructured energy levels and the emergence of new charge‐transfer pathways. The characteristic absorbance at 445 nm was used to determine the Ru(II) loading via a gradient dilution method (Figure S6), corresponding to a loading efficiency of approximately 39–49%. To corroborate this structural reorganization suggested by UV–vis spectral shifts, we turned to FT‐IR spectroscopy to probe metal‐ligand vibrations. As shown in Figure 1I, Cu‐ZIF8 displayed a characteristic metal‐N coordination band at ∼421.8 cm^−^^1^, consistent with the typical ZIF‐8 framework, alongside imidazole ring vibrations near 995 and 760 cm^−^^1^, confirming its structural integrity. In contrast, Cu‐ZIF8‐Ru exhibited systematic redshifts across key regions: the C = N (≈1500–1600 cm^−^^1^), C─N (≈1100–1300 cm^−^^1^), and metal‐N (<500 cm^−^^1^) stretching modes all shifted to lower wavenumbers. These collective shifts reflect decreased electron density at nitrogen sites and an altered coordination field, directly attributable to the electron‐withdrawing effect of the incorporated Ru(II) on the 2‐methylimidazole ligands, which lowers their vibrational frequencies. These FT‐IR observations are consistent with the electronic coupling indicated by UV–vis spectroscopy, collectively supporting the formation of a reconfigured heterometallic coordination environment in Cu‐ZIF8‐Ru. Critically, the metal‐N vibration red‐shifted from 421.8 cm^−^^1^ in Cu‐ZIF8 to 419.4 cm^−^^1^ in Cu‐ZIF8‐Ru (Figure S7), directly indicating a lowered vibrational frequency. This shift confirms the transformation of the original Cu─N coordination into new heterometallic vibrational modes. Together with the charge‐transfer pathways identified by UV–vis, these results provide compelling evidence for the formation of Ru and Cu coordination bonds that enable novel electronic coupling within the framework.

Although UV–vis and FT‐IR spectra indicated changes in electronic and vibrational states following Ru(II) incorporation, they fall short of identifying exact valence states or elucidating specific coordination‐electronic interactions. To elucidate these surface electronic and coordination effects, we conducted high‐resolution XPS analysis on Ru(II), Cu‐ZIF8, and Cu‐ZIF8‐Ru. In Cu‐ZIF8‐Ru, the Cu 2p peaks shifted to higher binding energies relative to Cu‐ZIF8 (Figure 1J), with increases of 0.2 eV for Cu 2p3/2 and 0.4 eV for Cu 2p1/2. These positive shifts indicate a depletion of electron density around Cu, consistent with an elevated effective oxidation state and a strengthened coordination field, confirming the electronic interaction between Cu and Ru. This positive shift is attributed to electron withdrawal from Cu by the adjacent Ru centers in the heterometallic structure. In marked contrast, the Zn 2p peaks remained virtually unchanged, with binding energies of 1021.6 eV (Zn 2p3/2) and 1044.6 eV (Zn 2p1/2) for Cu‐ZIF8, compared to 1021.7 and 1044.7 eV for Cu‐ZIF8‐Ru, respectively (Figure S8). The invariance of the Zn 2p signals indicates that the local electronic environment around Zn atoms was largely preserved upon Ru incorporation. This stark contrast demonstrates that Ru preferentially engages in electronic coupling through N‐coordination sites associated with Cu centers rather than Zn. The high‐resolution XPS spectrum of the free Ru(II) complex exhibited Ru3d doublet at 280.7 eV (Ru3d5/2) and 284.6 eV (Ru3d3/2) (Figure 1K). In Cu‐ZIF8‐Ru, only the Ru3d3/2 peak was detectable at 284.8 eV. The Ru3d5/2 signal is obscured due to overlap with the intense C 1s emission from the abundant carbon in the framework. Further evidence was provided by the Ru 3p XPS region. The binding energies for the Ru(II) complex are observed at 484.2 eV (Ru3p1/2) and 461.9 eV (Ru3p3/2). In comparison, the Ru3p1/2 peak in Cu‐ZIF‐8‐Ru appears at 483.7 eV, corresponding to a negative shift of 0.5 eV, while the Ru3p3/2 peak is located at 463.6 eV, reflecting a positive shift of 1.7 eV (Figure 1L). This shift signifies an elevated electron density around the Ru center. Furthermore, the spectrum of Cu‐ZIF8‐Ru revealed a new low‐energy satellite at ∼476.0 eV, a feature absent in the Ru(II) reference. The combination of the negative binding energy shift and the emerging satellite peak serves as direct spectroscopic evidence for Ru incorporation into unsaturated Cu‐N_3_ sites, ultimately leading to the formation of the Cu─N─Ru bridge structure. The N 1s XPS spectrum provided further evidence for electronic restructuring. In Cu‐ZIF8‐Ru, both the M─N and C─N peaks shifted to lower binding energies by 0.2 eV and 0.3 eV, respectively (Figure 1M), signifying increased electron density at nitrogen due to Ru coordination. These negative shifts on N, combined with the positive shift on Cu and the new spectral features on Ru, collectively confirm a Ru‐induced electron redistribution across an N‐bridged architecture, definitive of Cu─N─Ru bond formation.

### 2.2 Coordination Structure of Cu‐ZIF8‐Ru Nanozyme

**FIGURE 2:**
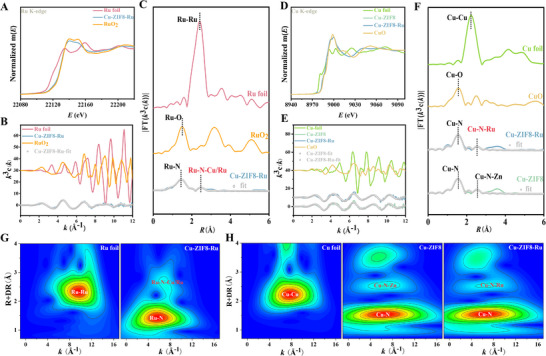
Synchrotron XAFS characterization of Cu‐ZIF8‐Ru nanozyme. (A) Normalized Ru K‐edge XANES spectra at the Ru K‐edge of Ru foil, RuO_2,_ and Cu‐ZIF8‐Ru. (B) k^3^‐weighted χ(k) oscillations at the Ru K‐edge for the Ru foil, RuO_2_, Cu‐ZIF8‐Ru and Cu‐ZIF8‐Ru‐fit. (C) Fourier‐transformed EXAFS spectra of the Ru K‐edge EXAFS spectra for the Ru foil, RuO_2_, Cu‐ZIF8‐Ru, and Cu‐ZIF8‐Ru‐fit at the Ru K‐edge. (D) Normalized Cu K‐edge XANES spectra of the Cu foil, CuO, Cu‐ZIF8, and Cu‐ZIF8‐Ru. (E) k^3^‐weighted χ(k) oscillations at the Cu K‐edge for Cu foil, CuO, Cu‐ZIF8, Cu‐ZIF8‐Ru, Cu‐ZIF8‐fit, and Cu‐ZIF8‐Ru‐fit. (F) Fourier‐transformed EXAFS spectra of the Cu K‐edge EXAFS spectra for the Cu foil, CuO, Cu‐ZIF8, Cu‐ZIF8‐Ru, Cu‐ZIF8‐fit, and Cu‐ZIF8‐Ru‐fit. (G) Wavelet transform (WT) contour plots of the Ru K‐edge EXAFS for Ru foil and Cu‐ZIF8‐Ru of the Ru K‐edge. (H) WT contour plots images of the Cu K‐edge EXAFS for Cu foil, Cu‐ZIF8, and Cu‐ZIF8‐Ru of the Cu K‐edge.

Atomic‐scale insights into the coordination environment and direct verification of the proposed Cu─N─Ru bridged sites were obtained through X‐ray absorption near‐edge structure (XANES) and extended X‐ray absorption fine structure (EXAFS) spectroscopy. Ru and Cu foils and their corresponding oxides were used as references. The Ru K‐edge XANES spectrum of Cu‐ZIF8‐Ru (Figure 2A; Figure S9A) displayed an absorption edge energy higher than that of Ru^0^ (foil) but lower than Ru^4^^+^ (RuO_2_). Furthermore, its white‐line intensity was intermediate between these two standards. These features collectively indicate that Ru in Cu‐ZIF8‐Ru exists in a formal oxidation state (Ru^δ^^+^, 0< δ < 4), ruling out the dominance of either metallic Ru clusters or fully oxidized RuO_2_‐like species. The Ru K‐edge EXAFS spectrum of Cu‐ZIF8‐Ru (Figure 2B) exhibited significantly attenuated oscillations and a phase shift compared to the reference foils and oxides, consistent with the theoretical fit. These spectral features are characteristic of a low‐coordination, highly disordered local environment around the Ru centers. The complete absence of these signals rigorously excludes the presence of metallic Ru clusters or extended Ru─O─Ru oxide domains, confirming that Ru is predominantly present as atomically dispersed sites. The Fourier‐transform (FT) EXAFS spectrum provided direct structural insight (Figure 2C). The dominant peak at ∼1.51 Å corresponds to the first‐shell Ru─N coordination. Notably, a distinct feature appears at ∼2.45 Å, which was identified through fitting as a multiple‐scattering path attributable to Ru─N─Cu/Ru. Critically, no peaks corresponding to Ru─Ru (∼2.39 Å in Ru foil) or Ru─O─Ru (∼1.53 Å in RuO_2_) scattering are present, excluding metallic or oxide phases. The definitive identification of this Ru─N─Cu/Ru scattering path provides direct spectroscopic evidence for an N‐bridged geometric linkage between Ru and Cu, confirming the formation of the proposed heterometallic Cu─N─Ru bridged active sites.

The Cu K‐edge XANES spectra revealed that the absorption edges of both Cu‐ZIF8 and Cu‐ZIF8‐Ru were between those of Cu^0^ (foil) and Cu^2^^+^ (CuO), with white‐line intensities comparable to CuO (Figure 2D; Figure S9B). This places the Cu species exist in a positive oxidation state (Cu^δ^^+^, 0< δ < 2), indicating that Ru incorporation does not fundamentally alter the average Cu oxidation state. However, subtle but distinct variations in the edge energy and spectral amplitude were observed between Cu‐ZIF8 and Cu‐ZIF8‐Ru, suggesting that Ru induces minor electronic and structural perturbations at the Cu sites. The Cu K‐edge EXAFS oscillations of both Cu‐ZIF8 and Cu‐ZIF8‐Ru (Figure 2E) showed strongly attenuated amplitudes relative to the references, in excellent agreement with theoretical fits. This pronounced damping, in excellent agreement with theoretical fits, is characteristic of a low‐coordination, disordered local environment, confirming the Cu─N coordination and the atomic dispersion of Cu without metallic or oxide phase contributions. In the Cu K‐edge FT‐EXAFS spectra (Figure 2F), the references Cu foil and CuO exhibited dominant peaks at ∼2.21 Å (Cu─Cu) and ∼1.56 Å (Cu─O), respectively. In contrast, both Cu‐ZIF8 and Cu‐ZIF8‐Ru showed a primary peak at ∼1.56 Å, assigned to first‐shell Cu─N coordination. A slight shift in this peak for Cu‐ZIF8‐Ru relative to Cu‐ZIF8 suggests Ru‐induced modulation of the Cu ligand field. Critically, no Cu─Cu metallic peak was observed in either framework material, ruling out Cu cluster formation. In the higher coordination shell, Cu‐ZIF8 featured a characteristic Cu─N─Zn multiple‐scattering path at ∼2.54 Å. In Cu‐ZIF8‐Ru, this feature was replaced by a Cu─N─Ru path at ∼2.57 Å, showing excellent agreement with the theoretical fit.

Although weak second‐shell peaks were observed for both Cu‐ZIF8 and Cu‐ZIF8‐Ru, their partial overlap in R‐space complicated unambiguous assignment based on amplitude alone. To resolve this ambiguity, we employed wavelet transform (WT)‐EXAFS analysis, which provides simultaneous resolution in both R‐ and k‐space. It leverages the fact that heavier scatterers like Ru yield stronger intensity at high k‐values, thus helping distinguish Cu─N─Zn from Cu─N─Ru paths. Distinct from the references, Cu‐ZIF8‐Ru exhibited a dominant Ru─N signal (R ≈ 1–2 Å, k ≈ 6–9 Å^−^^1^) and no evidence of Ru─Ru or Ru─O scattering at high k (Figure 2G; Figure S10A). Most importantly, a distinct intensity maximum at R ≈ 2.6–2.9 Å and k ≈ 7–10 Å^−^^1^ was identified, corresponding to a Ru─N─Cu/Ru multiple‐scattering path, providing direct evidence for heterometallic coupling. These WT‐EXAFS results conclusively show that Ru in Cu‐ZIF8‐Ru is exclusively coordinated by N in the first shell, with no detectable Ru─Ru or Ru─O neighbors. Furthermore, the identified multiple‐scattering path offers unambiguous evidence for an N‐bridged linkage between Ru and Cu. This atomic‐level structural insight is fully consistent with the electronic and vibrational perturbations revealed by UV–vis, FT‐IR, and XPS spectroscopy, collectively demonstrating the formation of the proposed Cu─N─Ru bridged active site. WT‐EXAFS analysis at the Cu K‐edge provided complementary structural validation. For Cu‐ZIF8, the spectrum (Figure 2H; Figure S10B) revealed a pronounced Cu─N first‐shell feature (R ≈ 1.3–1.8 Å, k ≈ 2–10 Å^−^^1^) and a distinct second‐shell Cu─N─Zn path (R ≈ 2.8–3.0 Å, k ≈ 6–9 Å^−^^1^), clearly different from the signals of Cu foil and CuO. In Cu‐ZIF8‐Ru, the strong Cu─N first‐shell feature persisted, confirming the integrity of the primary coordination geometry. Critically, however, the second‐shell signal appeared at R ≈ 2.8–3.0 Å but was shifted to higher k‐values (k ≈ 7–10 Å^−^^1^) with slightly enhanced intensity. This k‐space shift toward higher values is a direct signature of the heavier Ru backscatterer (compared to Zn), confirming the replacement of Zn by Ru in the second coordination shell and providing definitive evidence for the formation of heterobimetallic Cu─N─Ru bridges.

To obtain quantitative structural parameters and conclusively validate the heterobimetallic Cu─N─Ru bridge, we performed least‐squares EXAFS fitting on the Ru and Cu K‐edge data. For Cu‐ZIF8‐Ru, the Ru K‐edge data were satisfactorily fitted with two distinct first‐shell Ru‐N paths at 1.94 ± 0.01 Å (CN = 2.6 ± 0.5) and 2.09 ± 0.02 Å (CN = 2.5 ± 0.4) (Table S1). The second shell required the inclusion of both Ru─N─Cu (CN = 1.0 ± 0.2, R = 2.74 ± 0.02 Å) and Ru─N─Ru (CN = 2.0 ± 0.3, R = 2.74 ± 0.01 Å) multiple‐scattering paths for an optimal fit. The Ru─N─Cu path provides direct quantitative evidence from the Ru perspective for the N‐bridged coupling between Ru and Cu. The resolution of two distinct Ru─N bond lengths directly revealed an asymmetric first‐shell coordination environment around Ru. This structural asymmetry strongly corroborates the formation of the proposed asymmetric heterobimetallic Cu─N─Ru bridged site. Fitting of the Cu K‐edge EXAFS data demonstrated that the first‐shell Cu─N coordination in Cu‐ZIF8‐Ru (R = 1.97 ± 0.01 Å, CN ≈ 3.8) was nearly identical to that in Cu‐ZIF8 (Table S2). The pivotal change occurred in the second shell, where the Cu─N─Zn path in Cu‐ZIF8 (R = 2.89 ± 0.02 Å) was replaced by a Cu─N─Ru path in Cu‐ZIF8‐Ru at a shorter distance (R = 2.73 ± 0.03 Å). This shortening of the effective scattering distance confirms the formation of a more compact heterometallic bridge, aligning with the WT analysis. Collectively, the EXAFS fitting provides quantitative evidence that Ru is atomically dispersed and anchored via N‐bridging, successfully constructing the Cu‐ZIF8‐Ru nanozyme with defined Cu─N─Ru bridged sites.

### 2.3 Multiple Enzyme Activities of Cu‐ZIF8‐Ru Nanozyme

**FIGURE 3:**
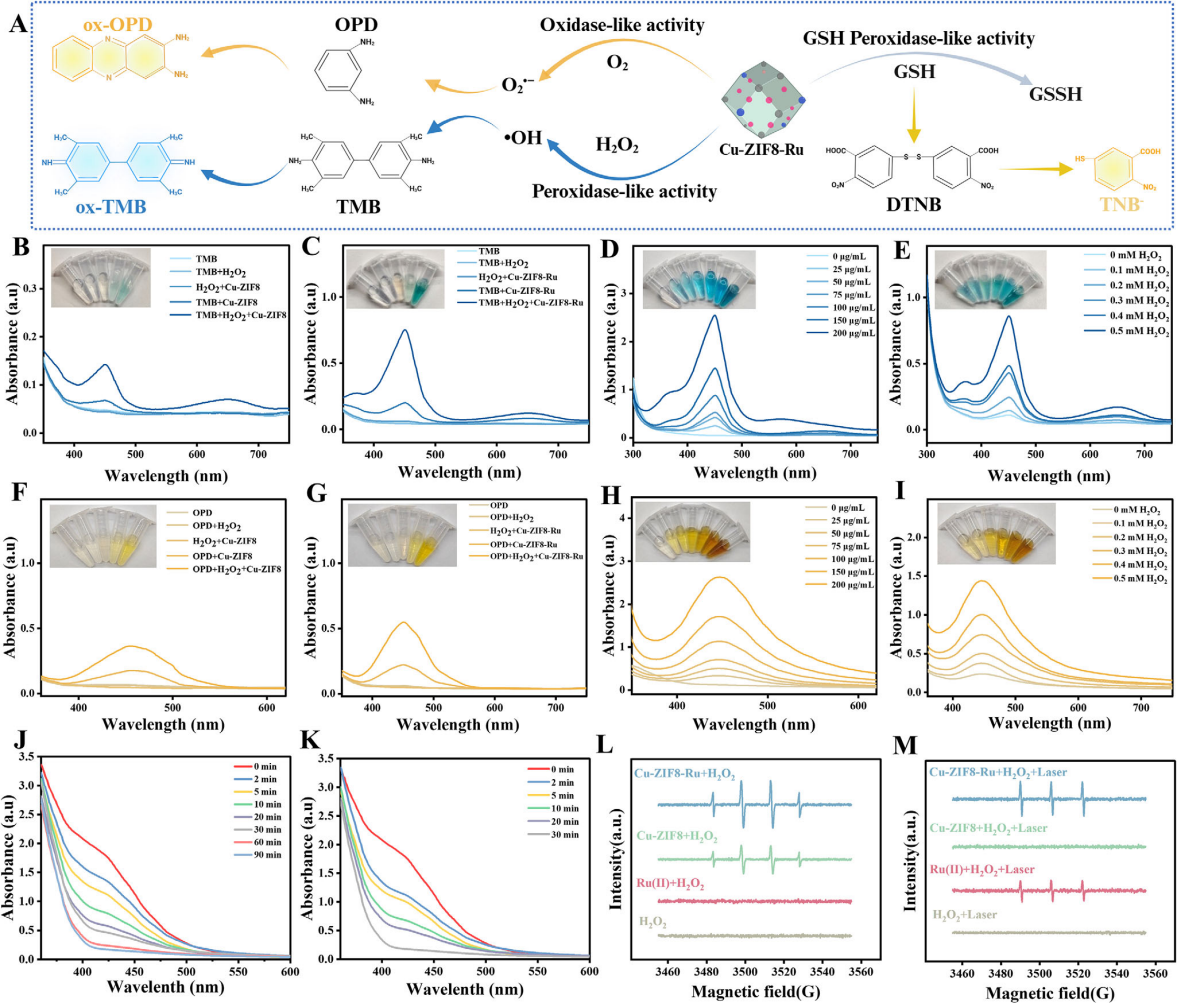
Enzyme‐like catalytic activities of Cu‐ZIF8‐Ru nanozyme. (A) Schematic illustration of the multienzyme activities of Cu‐ZIF8‐Ru nanozyme. (Created with BioRender.com) UV–vis spectra of the TMB oxidation assay in different treatments: (B) Cu‐ZIF8; (C) Cu‐ZIF8‐Ru. (D) POD‐like activity at varying Cu‐ZIF8‐Ru concentrations using the TMB probe. (E) POD‐like activity of Cu‐ZIF8‐Ru under increasing H_2_O_2_ concentrations (TMB probe). UV–vis spectra of the OPD oxidation assay: (F) Cu‐ZIF8; (G) Cu‐ZIF8‐Ru. (H) OXD‐like activity at varying Cu‐ZIF8‐Ru concentrations using the OPD probe. (I) OXD‐like activity of Cu‐ZIF8‐Ru under increasing H_2_O_2_ concentrations (OPD probe). Time‐dependent GSH depletion by Cu‐ZIF8‐Ru at 50 µg mL^−1^ (J) and 100 µg mL^−1^ (K) using the DTNB probe. ESR spectra of Cu‐ZIF8‐Ru detecting •OH (L) and ^1^O_2_ (M) generation under different conditions with or without Laser irradiation.

Asymmetric coordination geometries offer distinct advantages for enzyme mimetics, as their characteristic open metal sites and tunable ligand fields favor substrate adsorption and electron transfer, thus markedly enhancing catalytic performance [45]. To assess the multi‐enzyme mimetic activities of Cu‐ZIF8‐Ru were assessed by evaluating its peroxidase (POD)‐, oxidase (OXD)‐, and glutathione peroxidase (GSH‐Px)‐like activities were evaluated using 3,3′,5,5′‐tetramethylbenzidine (TMB), o‐phenylenediamine (OPD), and 5,5′‐dithiobis‐(2‐nitrobenzoic acid) (DTNB) as the respective chromogenic substrates. The corresponding catalytic mechanisms are illustrated in Figure 3A. To ensure comparability, all reactions were quenched simultaneously by adding a strong acid. As shown in Figure 3B, C, Cu‐ZIF8‐Ru oxidized TMB to blue oxTMB both in the presence and absence of H_2_O_2_. The addition of H_2_O_2_ produced a more intense color and stronger absorption peaks, demonstrating robust peroxidase (POD)‐like activity. Notably, significant TMB oxidation still occurred without H_2_O_2_, confirming its intrinsic oxidase (OXD)‐like activity. Furthermore, under identical conditions, Cu‐ZIF8‐Ru exhibited markedly stronger characteristic absorption peaks than Cu‐ZIF8, indicating enhanced OXD‐ and POD‐like activities. This enhancement is directly attributed to the heterobimetallic Cu─N─Ru bridged sites, which facilitate efficient electron transfer. As shown in Figure 3D, Cu‐ZIF8‐Ru oxidized TMB in the absence of H_2_O_2_, with the intensity of the resulting blue color increasing with nanozyme concentration, indicating concentration‐dependent OXD‐like activity. When the nanozyme concentration was fixed at 50 µg mL^−^^1^ (Figure 3E), both the color depth and the characteristic absorption peak intensity of the TMB solution increased at higher H_2_O_2_ concentrations, demonstrating H_2_O_2_‐dependent POD‐like activity. Furthermore, Cu‐ZIF8‐Ru effectively oxidized OPD to yellow oxOPD both with and without H_2_O_2_ (Figure 3F,G). The presence of H_2_O_2_ produced a deeper yellow color and stronger absorption peaks, indicating enhanced POD‐like activity. Owing to its high specific surface area and abundant active sites, Cu‐ZIF8‐Ru exhibited concentration‐dependent catalytic behavior with OPD that mirrored the trends observed with TMB under varying H_2_O_2_ and nanozyme concentrations (Figure 3H,I). Collectively, these results from both chromogenic substrates confirm that the Cu‐ZIF8‐Ru nanozyme possesses robust oxidase‐ and peroxidase‐like catalytic activities.

Given that depleting the bacterial antioxidant glutathione (GSH) represents a viable antibacterial strategy, we evaluated the glutathione peroxidase (GSH‐Px)‐like activity of Cu‐ZIF8‐Ru using the DTNB assay. In a system containing 50 µg mL^−^^1^ Cu‐ZIF8‐Ru and an equal concentration of GSH (Figure 3J), the absorbance at 412 nm decreased over time, indicating efficient GSH consumption and confirming GSH‐Px‐like activity. Notably, the characteristic peak decayed more rapidly for Cu‐ZIF8‐Ru than for Cu‐ZIF8 at the same concentration (Figure S11), demonstrating superior activity. This enhancement is likely due to the improved electron transfer facilitated by the heterobimetallic Cu─N─Ru bridged sites. Furthermore, at a concentration of 100 µg mL^−^^1^, Cu‐ZIF8‐Ru completely depleted GSH within 30 min (Figure 3K), demonstrating its potent and concentration‐dependent GSH‐Px‐like activity. Cu‐ZIF8‐Ru functions as a multifunctional nanozyme exhibiting robust oxidase‐, peroxidase‐, and glutathione peroxidase‐like activities, highlighting its great potential for synergistic antibacterial therapy. To identify the ROS central to the bactericidal effect, electron spin resonance (ESR) spectroscopy was conducted using 5,5‐dimethyl‐1‐pyrroline N‐oxide (DMPO) and 2,2,6,6‐tetramethylpiperidine (TEMP) as spin traps. Cu‐ZIF8‐Ru generated a stronger •OH signal than Cu‐ZIF8 under the same H_2_O_2_ concentration, as evidenced by the distinct 1:2:2:1 quartet (Figure 3L). Ru(II) as a photosensitizer for ^1^O_2_ generation under laser exposure has been extensively documented. Cu‐ZIF8‐Ru nanozyme exhibited a stronger ESR signal with a 1:1:1 triplet pattern under 660 nm laser irradiation compared to Ru(II) alone (Figure 3M), due to Ru(II) incorporated into Cu‐ZIF8 with a loading efficiency of 39–49%. This enhancement likely originates from the framework's high surface area and the Cu─N─Ru active sites, which promote electron transfer and mitigate charge recombination. Consequently, Cu‐ZIF8‐Ru outperformed its components in ROS generation. This culminates in a potent dual‐pathway antibacterial mechanism that uniquely synergizes exogenous ROS production with endogenous antioxidant depletion, positioning it as a highly effective nanozyme for synergistic therapy.

### 2.4 DFT Simulations of Catalytic Mechanism of Cu‐ZIF8‐Ru Nanozyme

**FIGURE 4:**
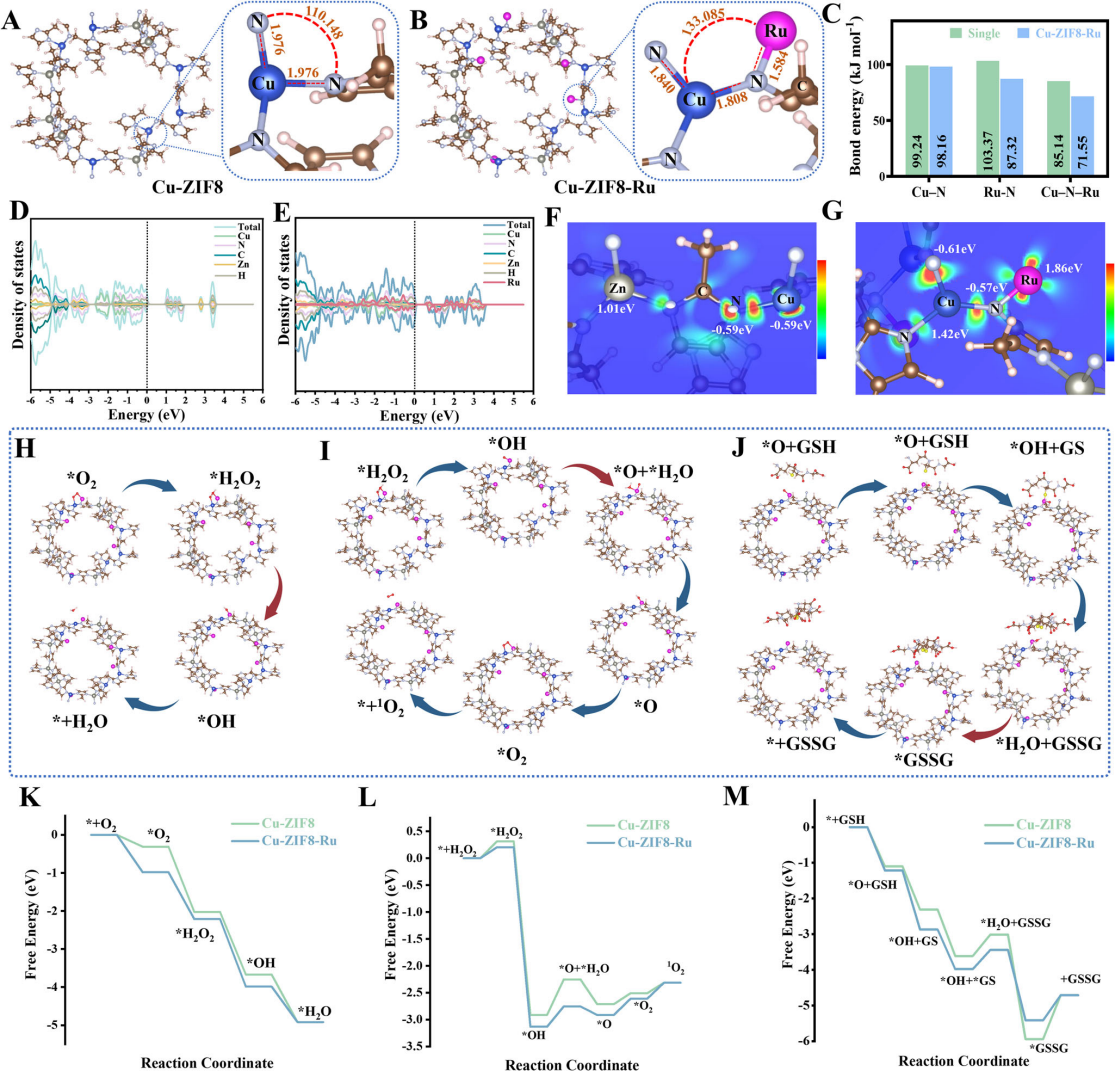
DFT simulations and proposed catalytic mechanism of Cu‐ZIF8‐Ru nanozyme. (A, B) Optimized structures of Cu‐ZIF8 and Cu‐ZIF8‐Ru. (C) The bond energies of Cu─N, Ru─N, and Cu─N─Ru. Total Density of states of (D) Cu‐ZIF8 and (E) Cu‐ZIF8‐Ru. (F) The charge density difference plots and the Bader charge distribution of Cu‐ZIF8 (the isosurface value is 0.002 e/Å3). (G) The charge density difference plots and the Bader charge distribution of Cu‐ZIF8‐Ru. DFT‐optimized intermediate structures and reaction pathways of (H) O_2_ reduction, (I) H_2_O_2_ decomposition, (J) GSH oxidation in Cu‐ZIF8‐Ru. Gibbs free energy diagrams for different pathways of (K) O_2_ reduction, (L) H_2_O_2_ decomposition, (M) GSH oxidation in Cu‐ZIF8‐Ru.

To gain atomic‐level insight into the mechanism underlying the enhanced nanozyme activity and ROS generation conferred by the heterobimetallic Cu─N─Ru bridge, we performed density functional theory (DFT) calculations. Structural models were constructed based on the ZIF‐8 framework and subsequently geometry‐optimized, with constraints guided by the experimental XAFS results. The geometry‐optimized structures of Cu‐ZIF8 and Cu‐ZIF8‐Ru are shown in Figure 4A,B, respectively. The DFT‐optimized structure of Cu‐ZIF8 revealed a typical unsaturated Cu─N_3_ coordination unit, with a Cu─N bond length of 1.976 Å and a bond angle of 110.148°. Incorporation of Ru leads to the formation of a heterobimetallic Cu─N─Ru bridged site, inducing a pronounced local geometric rearrangement. At this active site, the originally equivalent Cu─N bonds in Cu‐ZIF8 differentiated into two nonequivalent bonds of 1.808 and 1.840 Å. This bond‐length differentiation signals a strengthened ligand field at the Cu center and directly manifests the emergence of geometric asymmetry at the active site. Concurrently, the Ru─N bond length was calculated to be 1.584 Å, which is substantially shorter than the Cu─N bonds, reflecting stronger Ru─N coordination and tighter electronic coupling between the two metal centers. These computational findings are in excellent agreement with the experimental data from XAFS, XPS, and FT‐IR spectroscopy. Notably, the Cu─N─Ru bond angle increased to 133.085°, indicating a more open coordination geometry around the Cu center compared to the original Cu─N_3_ site. This asymmetric geometry facilitates both substrate binding/desorption and electron transfer. To further assess the structural stability of Cu‐ZIF8‐Ru (Figure 4C), we computed the binding energies of the Cu─N, Ru─N, and Cu─N─Ru bridged motifs. The computed bond energies for Cu‐ZIF8‐Ru were lower than those in the corresponding single‐site models, with the most pronounced decrease observed for the Cu─N─Ru bridge sites (from 85.14 to 71.55 kJ·mol^−^^1^). This optimized bond energy facilitates greater structural flexibility (bond vibration/angle opening) and electronic polarization during catalysis, translating into finely tuned reaction barriers and more efficient substrate conversion.

The modulation of the electronic structure by the asymmetric Cu─N─Ru bridge was probed through partial density of states (PDOS) calculations. Cu‐ZIF8 exhibited a very low total density of states (DOS) near the Fermi level (*E_F_*) with a pronounced pseudo‐gap (Figure 4D), arising primarily from weak hybridization between the Cu, N, and C orbitals, while contributions from Zn and H were negligible. This electronic structure suggests sparse states near *E_F_*, low charge carrier density, and consequently restricted charge transport. In contrast, Cu‐ZIF8‐Ru showed substantial filling and broadening of electronic states across the Fermi level, accompanied by a markedly elevated total DOS (Figure 4E). PDOS analysis revealed strong hybridization among Ru, Cu, and N orbitals near the Fermi level, which forms continuous electronic channels and shifts the d‐band center upward, most notably for Cu. These electronic modifications collectively demonstrate that constructing the Cu─N─Ru bridged sites enhances electrical conductivity, facilitates interfacial electron transfer, and ultimately lowers the reaction energy barrier. The regulation of the local electronic structure at the Cu─N─Ru bridge sites was further visualized through charge‐density difference plots and quantified by Bader charge analysis. As shown in Figure S12, the 3D charge‐density difference isosurfaces revealed that Cu‐ZIF8‐Ru exhibited more intense and continuous yellow/cyan isosurfaces around Cu─N─Ru bridge sites compared to Cu‐ZIF8. This indicates that Ru incorporation significantly enhanced electronic polarization and redistributed electron density toward the Cu─N─Ru bridge sites, identifying the heterobimetallic site as the central hub for nanozymatic catalysis. Analysis of the differential charge density near the Cu─N_3_ site in Cu‐ZIF8 revealed only weak polarization between the imidazolate ligand and the Cu center (Figure 4F), with limited charge redistribution on Cu and adjacent N atoms, indicating low polarizability. In contrast, Cu‐ZIF8‐Ru (Figure 4G) exhibited sheet‐like regions of electron accumulation and depletion around the bridging N atom. Furthermore, a clear directional charge redistribution was observed: the Ru side became electron‐rich, while the Cu side became electron‐deficient. The resulting polarized environment strengthens substrate adsorption/desorption and lowers the activation barrier for catalytic reactions on Cu‐ZIF8‐Ru. Bader charge analysis quantitatively confirmed this redistribution: the charge on Cu increased to +1.42 (from −0.59), Ru possessed a substantial positive charge (+1.86), and the bridging N maintained a slight negative charge (−0.57). This collectively indicates significant electron transfer within the Cu─N─Ru unit. These computational results align well with the XPS data, providing theoretical validation for the experimental observations. The asymmetric Cu─N─Ru bridge sites thus establish a directional electron‐transfer pathway and enhance electronic polarization, which collectively improve substrate binding dynamics, reduce reaction barriers, and ultimately elevate the nanozymatic catalytic efficiency.

To further elucidate the role of the asymmetric Cu─N─Ru bridge sites in enhancing the nanozymatic activity of Cu‐ZIF8‐Ru, we performed density functional theory (DFT) simulations to optimize the intermediate structures involved in its reactions with O_2_, H_2_O_2_, and GSH. Based on these optimized intermediates (Figure 4K–M; Figure S13), we proposed corresponding catalytic pathways and computed the associated Gibbs free‐energy changes. To mechanistically elucidate how the asymmetric Cu─N─Ru bridge sites enhance the nanozymatic activity of Cu‐ZIF8‐Ru, we performed density functional theory (DFT) simulations to optimize the structures of key reaction intermediates with O_2_, H_2_O_2_, and GSH. Based on the optimized intermediates (Figure 4K–M; Figure S13), we proposed corresponding catalytic pathways and computed the associated Gibbs free energy changes. The proton‐electron transfer sequences are summarized as follows: (1) * + O_2_ → *O_2_ + 2H^+^ + 2e^−^ → *H_2_O_2_ + H^+^ + e^−^ → *OH + H_2_O → *OH + H^+^ + e^−^ → *H_2_O → H_2_O + *; (2) * + H_2_O_2_ → *H_2_O_2_ + e^−^ → *OH + H^+^ + e^−^ → *O + *H_2_O → *O + O_2_ → *O_2_ → ^1^O_2_ + *; (3) * + GSH → *O + GSH → *OH + GS → *OH + *GS → *H_2_O + *GSSG → *GSSG + H_2_O → GSSG + *. The Gibbs free energy profiles (Figure 4K) reveal that Cu‐ZIF8‐Ru presents consistently lower energy barriers than Cu‐ZIF8 across key steps, particularly for O_2_→H_2_O_2_ and •OH→H_2_O conversions. This confirms that Ru incorporation facilitates O_2_ activation and electron transfer, directly accounting for the enhanced oxidase‐like activity and ROS generation observed experimentally without H_2_O_2_. In the H_2_O_2_ reaction pathway (Figure 4L), Cu‐ZIF8‐Ru exhibited lower energy barriers and more favorable energetics than Cu‐ZIF8 from *H_2_O_2_ adsorption through O─O cleavage to *O and *O_2_ formation, thermodynamically favoring ^1^O_2_ generation. Similarly, in the GSH pathway (Figure 4M), Cu‐ZIF8‐Ru displayed a smoother catalytic cycle with reduced barriers for hydrogen abstraction, cycle reset, and GSSG desorption. This overall enhancement in both pathways stems from the complementary electronic properties of the asymmetric Cu─N─Ru bridge sites: electron deficiency at Cu and electron enrichment at Ru jointly facilitate efficient electron transfer, thereby lowering the activation barriers. In summary, the enhanced nanozymatic activity of Cu‐ZIF8‐Ru originates from its asymmetric heterobimetallic Cu─N─Ru bridged sites. This unique configuration yields a more open coordination geometry and induces a polarized electronic environment: electron depletion around Cu alongside electron enrichment on Ru promotes charge separation. Concurrently, an upshifted d‐band center toward the Fermi level facilitates interfacial electron transfer and lowers activation barriers. These effects are synergistically amplified by a directional Cu → N → Ru electron‐transfer channel, which intensifies electronic polarization and optimizes the adsorption/desorption of reaction intermediates, thereby significantly boosting the overall catalytic efficiency.

### 2.5 Evaluation of the Bactericidal Capacity of Cu‐ZIF8‐Ru Nanozyme

The multi‐enzyme activities of Cu‐ZIF8‐Ru, which enable synergistic ROS generation and disruption of bacterial antioxidant defenses, support its potential for treating infections. Given that infection sites are often mildly acidic (pH ≈ 5.5–6.5), we assessed the acid‐responsive stability of Cu‐ZIF8‐Ru. As shown in Figure S14, at pH 7.4, Cu‐ZIF8‐Ru maintained a well‐defined polyhedral morphology. At pH 6.5, particle edges became blunted with visible surface corrosion and etch pits, accompanied by a more rounded overall form. At pH 5.5, severe structural dissolution and collapse occurred, yielding irregular, nearly spherical particles with coarse surfaces. Additionally, the POD‐like activity of the Cu‐ZIF8‐Ru nanozyme after incubation in an acidic environment for varying durations was assessed using the TMB probe method. The results revealed a slight decline in POD‐like activity with increasing incubation time in the acidic solution (Figure S15). This indicates that its ROS‐generating capacity becomes gradually diminished upon prolonged exposure to acidic conditions. This progressive morphological degradation corresponds to pH‐dependent pore opening and catalytic site exposure, thereby facilitating the controlled release of metal ions under acidic conditions. More importantly, this self‐limiting disintegration within the infectious microenvironment ensures biosafety: it restricts the prolonged persistence of active sites and excessive ROS generation, effectively preventing sustained oxidative damage to surrounding healthy tissues. Prior to considering clinical translation, especially for urinary tract applications, the biocompatibility of Cu‐ZIF8‐Ru was systematically evaluated. The viability of normal human urothelial cells (SV‐HUC‐1) remained high (90%–100%) after exposure to Cu‐ZIF8‐Ru across a wide concentration range (0–400 µg mL^−^^1^) and various Cu loadings (10%–30%), demonstrating no significant cytotoxicity (Figure S16A). In contrast, human umbilical vein endothelial cells (HUVECs) exhibited dose‐dependent growth inhibition at concentrations above 200 µg mL^−^^1^, an effect slightly enhanced by higher Cu content (Figure S16B). This differential sensitivity is likely due to the higher basal metabolic activity and greater susceptibility to ROS of HUVECs. To strike an optimal balance between efficacy and safety, the 20% Cu formulation was chosen for antibacterial assays within a 0–200 µg mL^−^^1^ range. The viability of SV‐HUC‐1 cells across this concentration range was assessed using the CCK‐8 assay. To evaluate potential phototoxicity, separate cell groups were exposed to the treatments with or without laser irradiation (660 nm, 100 mW cm^−^^2^, 10 min). For comparative purposes, the effects of Ru(II), Cu‐ZIF8, and Cu‐ZIF8‐Ru on cell viability were systematically evaluated over the same concentration range (0–200 µg mL^−^^1^), with the results presented in Figure S16C,D. Both in the dark and under laser irradiation, Cu‐ZIF8‐Ru exhibited excellent biocompatibility with SV‐HUC‐1 cells at concentrations up to 200 µg mL^−^^1^, with no significant cytotoxicity observed. Hemocompatibility assays further confirmed its clinical safety potential (Figure S17), showing a hemolysis rate below 3% across 50–400 µg mL^−^^1^, which is within the accepted safety threshold (<5%). These results collectively demonstrate that Cu‐ZIF8‐Ru possesses a favorable biosafety profile suitable for intravesical instillation in MDR‐UTIs treatment.

**FIGURE 5:**
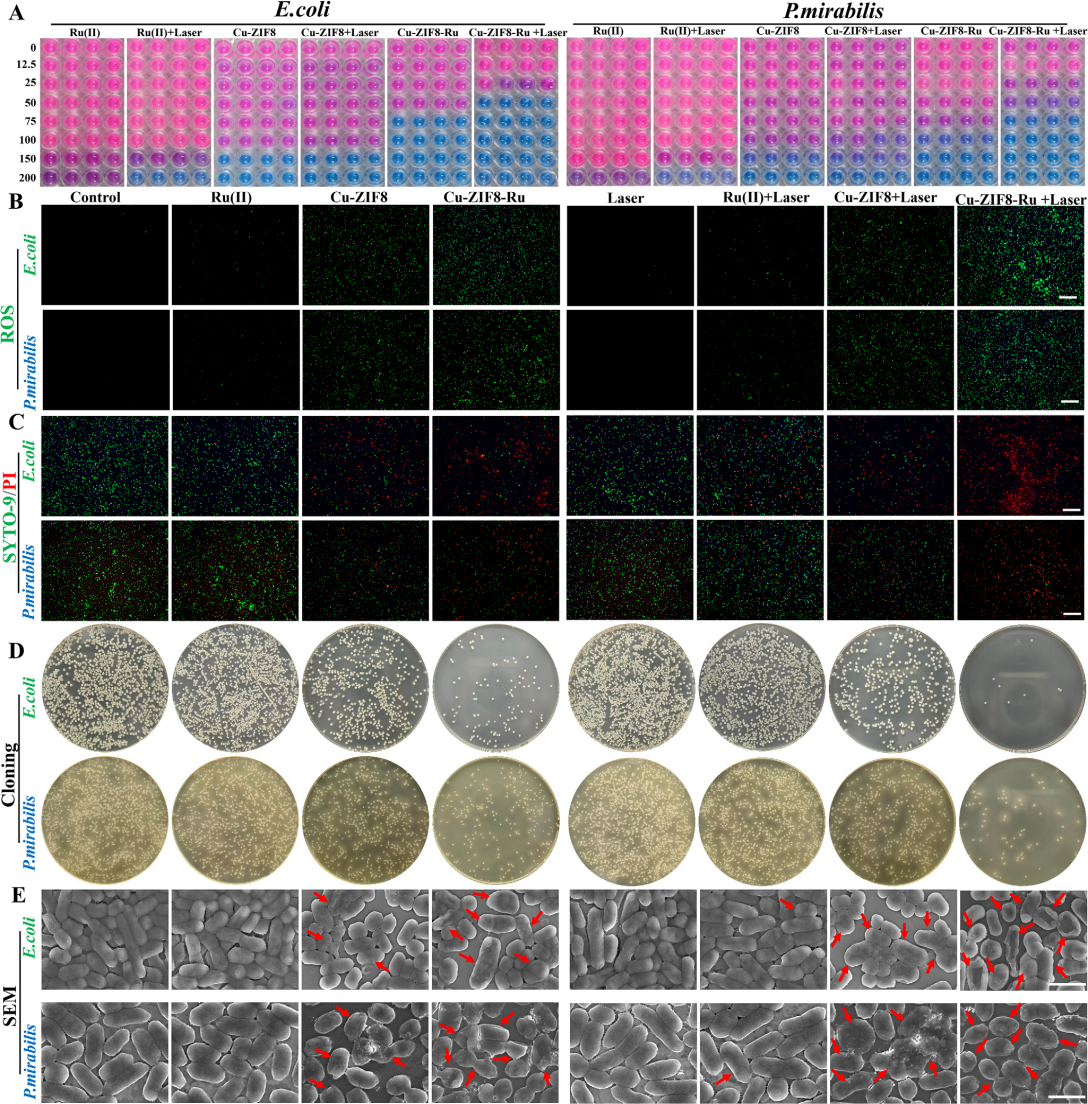
The Bactericidal effects of Cu‐ZIF8‐Ru nanozyme against MDR *E. coli* and MDR *P. mirabilis*. (A)The antibacterial activity of Cu‐ZIF8‐Ru against MDR *E. coli* and MDR *P. mirabilis* in different treatments and different concentrations was detected by Alamar Blue reagent. (B) ROS generation in MDR *E. coli* and MDR *P. mirabilis* detected by DCFH‐DA fluorescence staining (scale bar = 200 µm). (C) Bacterial viability determined by SYTO 9 (live, green) and propidium iodide (dead, red) double staining (scale bar = 200 µm). (D) Colony formation assay of MDR *E. coli* and MDR *P. mirabilis* in different treatments. (E) SEM images of MDR *E. coli* and MDR *P. mirabilis* displaying morphological damage after treatment (representative defects marked by red arrows, scale bar = 5 µm).

To systematically evaluate the antibacterial activity of the Cu‐ZIF8‐Ru nanozyme (Table S3), we employed clinical urinary tract isolates of multidrug‐resistant Escherichia coli (MDR *E. coli*) and multidrug‐resistant Proteus mirabilis (MDR *P. mirabilis*) as model pathogens. Bacterial viability was quantified using the Alamar Blue assay, wherein metabolic reduction of the blue, non‑fluorescent dye to a pink, fluorescent product by living bacteria provides a direct measure of bacterial survival. As shown in Figure S18, neither MDR *E. coli* nor MDR *P. mirabilis* showed statistically significant differences in quantitative fluorescence readings across the four tested conditions (pH 7.4, pH 5.5, pH 7.4 + H_2_O_2_, and pH 5.5 + H_2_O_2_), confirming that the incubation conditions alone had no substantial effect on bacterial viability. We therefore systematically evaluated the antibacterial activity of Cu‐ZIF8‐Ru against MDR *E. coli* under combinations of these varying conditions using the Alamar Blue assay. As shown in Figure S19A, the color of the assay wells shifted from pink to blue‐purple with increasing concentrations of Cu‐ZIF8‐Ru, indicating a marked, dose‐dependent antibacterial effect. At any given concentration, the blue‐purple color was more intense at pH 5.5 than at pH 7.4, demonstrating enhanced activity in an acidic microenvironment. The addition of H_2_O_2_ or application of laser irradiation alone further deepened the color, and their combination resulted in the most profound blue‐purple hue, indicating a potent synergistic antibacterial effect. These qualitative observations were corroborated by quantitative analysis (Figure S19B,C), which substantiated that the dose‐dependent antibacterial activity of Cu‐ZIF8‐Ru against MDR *E. coli* was significantly enhanced by acidic pH, H_2_O_2_, and laser irradiation. Based on these findings, we designed an eight‐group experiment to systematically deconvolute and compare the individual contributions of each factor to the antibacterial efficacy against both MDR *E. coli* and MDR *P. mirabilis*. As shown in Figure 5A, the color of the assay wells transitioned from pink to blue‐purple with increasing concentrations of Cu‐ZIF8‐Ru for both bacterial strains, indicating a shift from metabolic activity to inhibition and cell death. In the absence of laser irradiation, Cu‐ZIF8‐Ru exhibited significantly greater antibacterial efficacy than either Cu‐ZIF8 or Ru(II) alone. Laser irradiation markedly enhanced the antibacterial activity, and the Cu‐ZIF8‐Ru + Laser group showed the most potent effect with a clear dose‐response. The MIC for MDR *E. coli* (50 µg mL^−^^1^) was lower than that for MDR *P. mirabilis* (75 µg mL^−^^1^) (Figures S20 and S21), indicating the former's heightened sensitivity. This broad‐spectrum efficacy was further corroborated by fluorescence quantification.

To further evaluate antibacterial efficacy, we compared different agents at the same concentration (50 µg mL^−^^1^, quantified by Cu‐ZIF8‐Ru). Intracellular ROS generation was first monitored using the fluorescent probe DCFH‐DA. As shown in Figure 5B, negligible fluorescence was detected in the control and laser‐only groups. Ru(II) and Cu‐ZIF8 alone produced limited green fluorescence, while Cu‐ZIF8‐Ru alone induced a markedly stronger signal. Upon laser irradiation, the Cu‐ZIF8‐Ru + Laser group exhibited the most intense fluorescence in both bacterial strains, surpassing that of the Ru(II) + Laser and Cu‐ZIF8 + Laser groups. This demonstrates that the asymmetric heterobimetallic Cu‐N─Ru bridge in Cu‐ZIF8‐Ru triggers a more potent intracellular ROS burst. Notably, MDR *E. coli* was more sensitive to Cu‐ZIF8‐Ru than MDR *P. mirabilis*, a difference accompanied by higher intracellular ROS levels and confirmed by quantitative data (Figure S22A,B). Bacterial membrane integrity and viability in response to Cu‐ZIF8‐Ru treatment were then assessed for both strains using the SYTO9/PI double‐staining assay. As shown in Figure 5C, microscopic fields in the control and laser‐only groups were predominantly green, indicating that most bacterial cells remained intact and viable. Treatment with Ru(II) or Cu‐ZIF8 alone resulted in only scattered red fluorescence, suggesting limited membrane damage and bactericidal effect. In contrast, Cu‐ZIF8‐Ru alone significantly increased red fluorescence, demonstrating substantial membrane disruption even without irradiation. Most notably, the Cu‐ZIF8‐Ru + Laser group showed the strongest effect: green fluorescence was largely replaced by intense, confluent red staining in both strains, indicating extensive membrane damage and irreversible cell death. These results confirm that Cu‐ZIF8‐Ru effectively compromises bacterial membrane integrity, that MDR *E. coli* is more susceptible than MDR *P. mirabilis*, and that laser irradiation synergistically amplifies this bactericidal effect. As a quantitative complement to fluorescence staining, colony formation assays were conducted to evaluate bacterial viability. Control and laser‐only groups showed confluent growth (Figure 5D), while Ru(II) or Cu‐ZIF8 caused a minor reduction. Cu‐ZIF8‐Ru alone strongly inhibited both strains, with a greater effect on MDR *E. coli*. In combination with laser irradiation, it nearly eradicated MDR *E. coli* and markedly reduced MDR *P. mirabilis*, confirming potent, strain‐dependent bactericidal action. Quantitative colony counts (Figure S22C,D) further validated these results. To investigate the effect of Cu‐ZIF8‐Ru on bacterial envelope integrity, we performed SEM on treated bacteria. SEM directly visualized the progressive damage to bacterial morphology (Figure 5E). Untreated and laser‐only controls of both MDR *E. coli* and MDR *P. mirabilis* retained their characteristic rod shape with smooth, intact surfaces, indicating preserved envelope integrity. Treatment with Ru(II) or Cu‐ZIF8 alone induced only sporadic surface wrinkling and minor depressions, consistent with limited injury. In contrast, Cu‐ZIF8‐Ru alone caused markedly more severe damage, characterized by abundant pits, grooves, and localized surface collapse. Many bacteria exhibited membrane blebbing and deformation, suggesting increased permeability and cytoplasmic leakage. Notably, in the Cu‐ZIF8‐Ru + Laser group, structural damage was most severe, featuring widespread membrane tearing and profound surface corrugation. These morphological alterations collectively confirm the irreversible, laser‐amplified disruption of the bacterial envelope.

Bacterial biofilms are self‐protective, multicellular communities embedded in an extracellular polymeric matrix, which confer formidable resistance to conventional antibiotics. Their eradication therefore, constitutes a major therapeutic hurdle. To address this challenge, the anti‐biofilm activity of Cu‐ZIF8‐Ru against MDR *E. coli* was investigated and compared to other treatments. Crystal violet staining was employed to assess both the inhibition of biofilm formation and the disruption of pre‐formed biofilms. In the inhibition assay (Figure S23A,B), treatment with Ru(II) or laser irradiation alone demonstrated no significant effect, while Cu‐ZIF8 provided partial suppression. Cu‐ZIF8‐Ru was substantially more effective, and its combination with laser irradiation resulted in near‐colorless wells, indicating near‐total suppression of biofilm formation; quantification confirmed this superior efficacy. For the disruption of mature biofilms (Figure S23C,D), neither Ru(II) alone nor laser irradiation alone substantially reduced the staining intensity compared to the control. Cu‐ZIF8 treatment led to a moderate reduction, suggesting partial biofilm disintegration. In contrast, Cu‐ZIF8‐Ru alone caused a profound reduction in staining even without irradiation. When combined with laser irradiation, the biofilm staining was almost completely eradicated, indicating thorough structural disintegration of the mature biofilm. A consistent antibacterial trend was observed against MDR *P. mirabilis* biofilms. In the biofilm formation inhibition assay, the control, laser‐only, and Ru(II)‐only groups were largely ineffective, while Cu‐ZIF8 provided only modest suppression (Figure S24A,B). Conversely, Cu‐ZIF8‐Ru alone induced pronounced inhibition, significantly reducing biofilm staining; this effect was maximized under laser irradiation, achieving the highest inhibition rate. Similarly, in the biofilm disruption assay (Figure S24C,D), Cu‐ZIF8‐Ru attenuated mature biofilms even without irradiation and exhibited markedly enhanced clearance with irradiation, decisively outperforming its individual components. These results indicate that Cu‐ZIF8‐Ru nanozymes induce potent bacterial inhibition and killing by triggering intense intracellular ROS bursts and damaging the bacterial envelope.

### 2.6 The Bactericidal Mechanism of Cu‐ZIF8‐Ru Nanozyme

**FIGURE 6:**
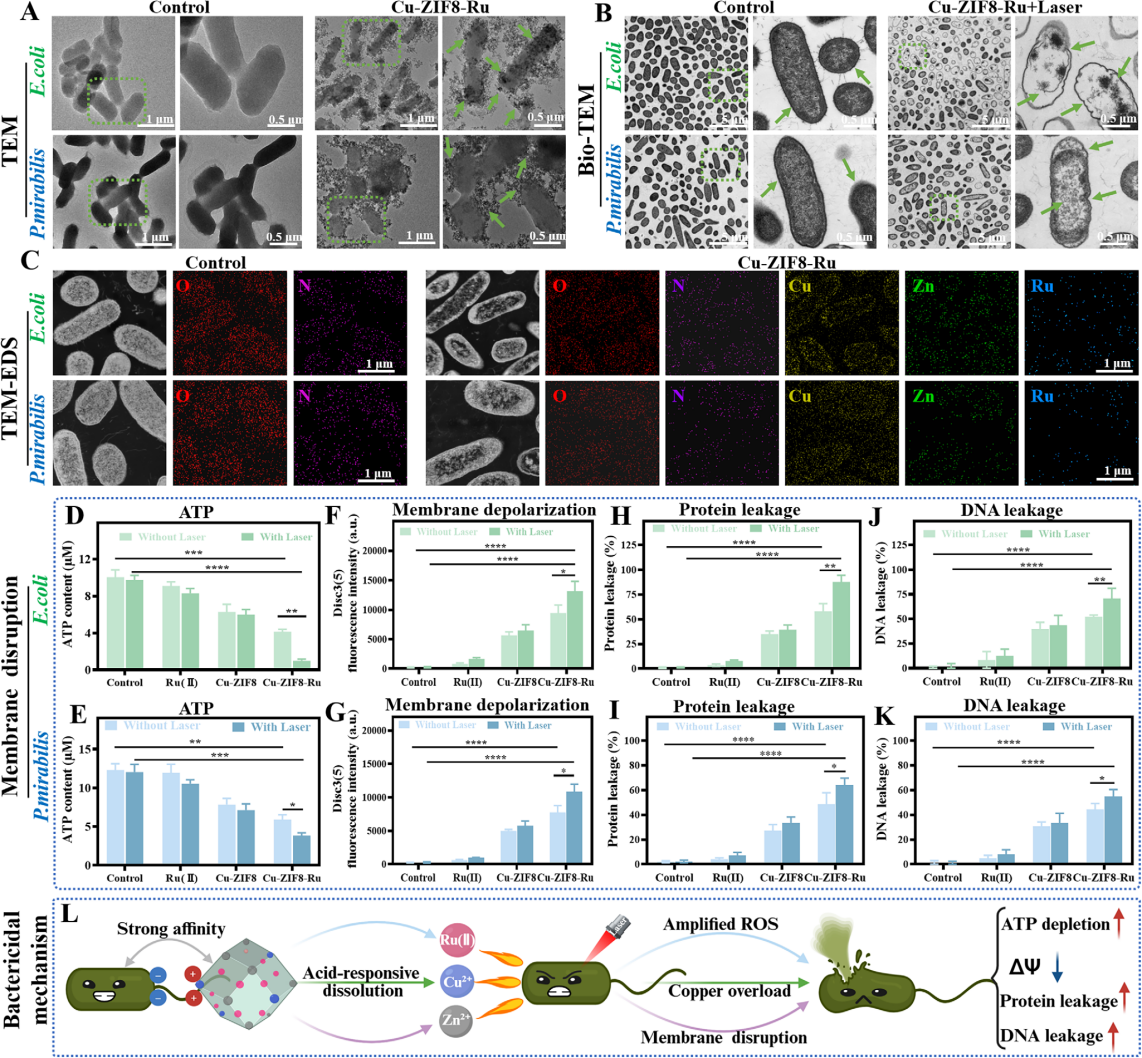
The Bactericidal mechanism of Cu‐ZIF8‐Ru nanozyme. (A) TEM images of MDR *E. coli* and MDR *P. mirabilis* after Cu‐ZIF8‐Ru treatment. (B) Bio‐TEM images of MDR *E. coli* and *P. mirabilis* after Cu‐ZIF8‐Ru treatment. (C) TEM‐EDS mapping of MDR *E. coli* and MDR *P. mirabilis* after Cu‐ZIF8‐Ru treatment. (D–K) Quantitative assessment of key functions in MDR *E. coli* (D, F, H, J) and MDR *P. mirabilis* (E, G, I, K). (L) Schematic illustration of the antibacterial mechanism of Cu‐ZIF8‐Ru. (Created with BioRender.com) Data in panels (D–K) are expressed as mean ± SD (n = 3). Error bars represent SD.

Following the confirmation of the pronounced bactericidal effects of Cu‐ZIF8‐Ru against MDR *E. coli* and MDR *P. mirabilis*, we used TEM to investigate its interaction with the bacterial cells. As shown in Figure 6A, control bacteria of both strains exhibited pristine, rod‐shaped morphologies with smooth surfaces. In contrast, upon treatment with Cu‐ZIF8‐Ru, a dense adherence of nanozyme particles was observed on the bacterial surfaces. This targeted binding is likely mediated by electrostatic attraction between the positively charged nanozyme and the negatively charged components of the bacterial envelope. To visualize these damages at the ultrastructural level, we performed bio‐transmission electron microscopy (bio‐TEM) on MDR *E. coli* and MDR *P. mirabilis* following Cu‐ZIF8‐Ru treatment (Figure 6B). Control bacteria of both strains exhibited intact morphology with electron‐dense cytoplasm and continuous, well‐defined bacterial envelopes. In contrast, treated bacteria displayed a constellation of severe ultrastructural damages, including membrane undulation and rupture, cytoplasmic rarefaction, and vacuolation. These alterations provide direct evidence of critical barrier failure and leakage of intracellular contents. Notably, the destructive effects were more extensive in MDR *E. coli* than in MDR *P. mirabilis*. Following the disruption of the cell envelope, Zn^2^^+^ and Cu^2^^+^ ions released from the acid‐degraded Cu‐ZIF8‐Ru nanozyme enter the bacterial cytosol. The spatial distribution of metal ions associated with the bacteria was investigated using TEM‐EDS (Figure 6C). In control samples, elemental mapping detected only homogeneous O and N signals from cellular constituents, with no exogenous metal signals. For Cu‐ZIF8‐Ru‐treated MDR *E. coli* and MDR *P. mirabilis*, intense Cu signals were observed, displaying a peripheral distribution that traced the bacteria outline. Zn signals were similarly localized to the cell margins, and Ru signals co‐localized with Cu and Zn at the bacterial surface. These observations indicate that disruption of the bacterial envelope by Cu‐ZIF8‐Ru is followed by accelerated entry of Zn^2^^+^ and Cu^2^^+^ into the cytosol, resulting in intracellular copper overload. Bacterial membrane integrity was further assessed using the fluorescent probe propidium iodide (PI). As summarized in Figure S25, both bacterial strains exhibited minimal fluorescence in the control and Ru(II) groups. Treatment with Cu‐ZIF8 induced a moderate increase, whereas Cu‐ZIF8‐Ru generated the most pronounced fluorescence signal. This signal was further enhanced under laser irradiation. These results confirmed that Cu‐ZIF8‐Ru potently disrupts the membrane of MDR *E. coli* and MDR *P. mirabilis*, thereby corroborating the membrane damage visualized in the earlier morphological studies.

To evaluate the functional consequences of membrane damage, we assessed key indicators of metabolic and physiological integrity: intracellular ATP levels, membrane potential, and leakage of proteins and DNA. Assessment of intracellular ATP revealed that Cu‐ZIF8 treatment reduced ATP levels relative to controls (Figure 6D,E). In contrast, Cu‐ZIF8‐Ru induced a far greater reduction, and the Cu‐ZIF8‐Ru + Laser group exhibited the lowest ATP levels. Most notably, this severe energy depletion underscores a critical compromise of bacterial energetics, leading to metabolic collapse. Membrane depolarization is an early indicator of envelope injury. Cu‐ZIF8‐Ru treatment induced a greater increase in DiSC3(5) fluorescence relative to controls and the Cu‐ZIF8 group (Figure 6F,G), and the signal was further augmented by laser irradiation, signifying the most severe Membrane depolarization collapse. This confirms that Cu‐ZIF8‐Ru induces membrane depolarization and permeabilization, thereby facilitating enhanced intracellular metal accumulation. To evaluate the extent of envelope damage, we measured the leakage of proteins and DNA, a direct and consequential indicator of membrane integrity. As shown in Figure 6H,I, the most pronounced protein leakage was observed in the Cu‐ZIF8‐Ru group, the effect that was far more substantial under laser irradiation and significantly exceeded all other treatments. This confirms a substantial breach in membrane permeability. A similar trend was observed for DNA leakage (Figure 6J,K), reinforcing the conclusion of severe membrane disruption. As summarized in Figure 6L, the antibacterial action of Cu‐ZIF8‐Ru involves electrostatic targeting and acid‐triggered release of Cu^2^^+^/Zn^2^^+^ and Ru(II). Laser irradiation dramatically amplifies ROS generation, which synergizes with the released metal ions to disrupt the cell envelope. This damage promotes further intracellular Cu^2^^+^ accumulation, resulting in membrane potential collapse, ATP depletion, and leakage of proteins and DNA. This multi‐faceted attack creates a synergistic cycle of damage that causes irreversible metabolic and structural failure in MDR *E. coli* and MDR *P. mirabilis*, with efficacy significantly boosted by laser activation.

### 2.7 Cu‐ZIF8‐Ru Nanozyme Induced Cuproptosis of Multidrug‐Resistant Bacteria

**FIGURE 7:**
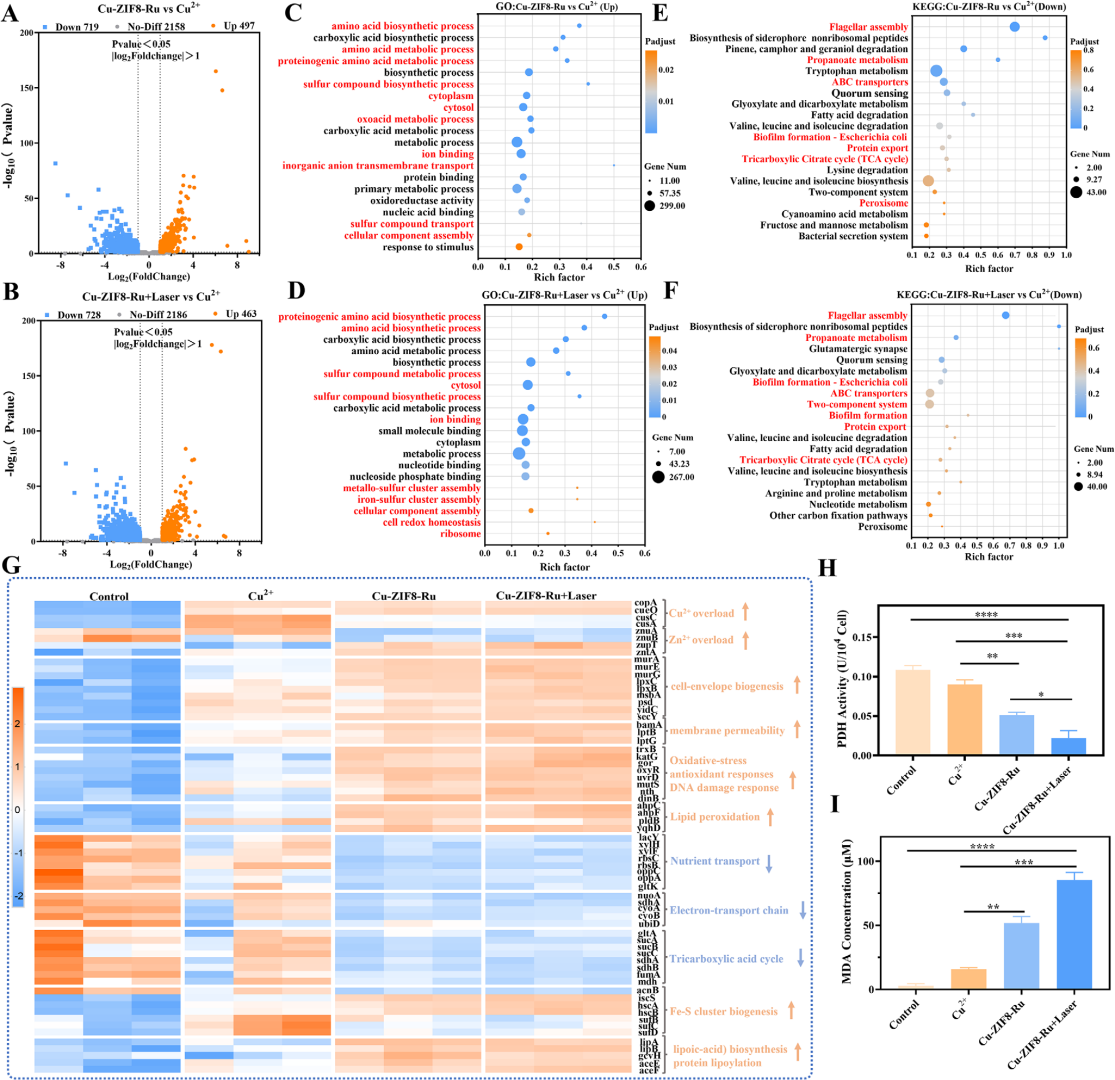
RNA sequencing analysis of the antibacterial mechanism of Cu‐ZIF8‐Ru nanozyme. (A, B) Volcano plots of DEGs for the comparisons of (A) Cu‐ZIF8‐Ru vs Cu^2^^+^ and (B) Cu‐ZIF8‐Ru+Laser vs Cu^2^^+^. (C) GO enrichment analysis of upregulated DEGs in Cu‐ZIF8‐Ru vs Cu^2^^+^. (D) GO enrichment analysis of upregulated DEGs in Cu‐ZIF8‐Ru+Laser vs Cu^2^^+^. (E) KEGG enrichment analysis of downregulated DEGs in Cu‐ZIF8‐Ru vs Cu^2^^+^. (F) KEGG enrichment analysis of downregulated DEGs in Cu‐ZIF8‐Ru+Laser vs Cu^2^^+^. (G) The heatmap shows the upregulation or downregulation of representative DEGs related to Cu^2^^+^/Zn^2^^+^ overload, cell envelope biogenesis, oxidative stress, DNA damage, lipid peroxidation, nutrient transport, electron transport chain, TCA cycle, Fe─S cluster biogenesis, lipoic acid, biosynthesis, and protein lipoylation. (H) PDH activity of MDR *E. coli* after incubation with Cu^2+^ and Cu‐ZIF8‐Ru (10 µg/mL, Cu^2+^ equivalent) measured by the PDH activity assay kit. (I) MDA concentration in MDR *E. coli* after incubation with Cu^2+^ and Cu‐ZIF8‐Ru (10 µg/mL, Cu^2+^ equivalent) measured by the MDA assay kit. Values represent the mean ± SD from three independent experiments (H, I). Error bars indicate SD.

Copper is an essential micronutrient for bacteria, playing critical roles as a cofactor in cuproenzymes involved in central metabolism and respiration. While bacteria employ dedicated membrane transporters to maintain intracellular copper homeostasis, dysregulation and overload can trigger lethal toxicity. To systematically decipher the antibacterial mechanism of Cu‐ZIF8‐Ru at the molecular level, we performed RNA sequencing on MDR *E. coli* treated with free Cu^2^^+^ ions, Cu‐ZIF8‐Ru alone, or its laser‐activated counterpart. This approach allowed us to identify the specific gene expression alterations and pathways underlying the nanozyme's efficacy. Principal component analysis (PCA) revealed clear separation among the Control, Cu^2^^+^, Cu‐ZIF8‐Ru, and Cu‐ZIF8‐Ru+Laser groups along the first two principal components (PC1: ∼62.8%; PC2: ∼17.0%) (Figure S26). The Control samples clustered in the lower‐right quadrant, while the Cu^2^^+^ group was positioned distinctly from the Cu‐ZIF8‐Ru groups. Notably, both Cu‐ZIF8‐Ru and Cu‐ZIF8‐Ru+Laser treatments clustered closely in the lower‐left quadrant, distant from other groups, indicating a pronounced and coherent transcriptomic reprogramming specific to the nanozyme treatment. Comparative analysis of differentially expressed genes (DEGs) showed that, relative to the Cu^2^^+^ vs Control comparison (Figure S27A), the Cu‐ZIF8‐Ru (Figure S27B) and Cu‐ZIF8‐Ru+Laser (Figure S27C) treatments induced substantially more DEGs. This indicates that while free Cu^2^^+^ elicits a defined stress response, Cu‐ZIF8‐Ru exerts a broader transcriptomic impact, which is further intensified by laser irradiation. Furthermore, a large number of DEGs were identified when comparing either Cu‐ZIF8‐Ru (Figure 7A) or Cu‐ZIF8‐Ru+Laser (Figure 7B) against the Cu^2^^+^ group. In contrast, the direct comparison between Cu‐ZIF8‐Ru+Laser and Cu‐ZIF8‐Ru (Figure S27D) yielded only a limited set of DEGs, suggesting that photoactivation primarily amplifies pre‐existing response pathways rather than inducing entirely new ones. Collectively, these transcriptomic profiles demonstrate that Cu‐ZIF8‐Ru markedly augments and expands the regulatory influence of Cu^2^^+^ on MDR *E. coli*, with the broadest antibacterial action observed under laser. Based on these transcriptomic findings, GO and KEGG enrichment analyses were performed on the DEGs. The analysis focused on metal‐ion response, redox homeostasis, cell‐envelope biogenesis, and energy metabolism pathways to delineate the synergistic contributions of ROS burst and Cu^2^^+^ overload to the bactericidal mechanism.

GO enrichment analysis was performed on the DEGs identified in the Cu‐ZIF8‐Ru vs Cu^2^^+^ (Figure S28A) and Cu‐ZIF8‐Ru+Laser vs Cu^2^^+^ (Figure S28B) comparisons to delineate the key biological processes affected. In the Cu‐ZIF8‐Ru vs Cu^2^^+^ comparison, upregulated DEGs were primarily associated with protein and amino acid metabolism, carboxylic acid metabolism, biosynthetic processes, sulfur‐containing compound biosynthesis, and cytoplasmic pathways (Figure 7C). Enrichment was also observed in inorganic anion transmembrane transport, ion binding, and oxidoreductase activity. These findings indicate that, relative to free Cu^2^^+^, Cu‐ZIF8‐Ru intensifies metal and oxidative stresses in MDR *E. coli*, triggering broad metabolic compensation and stress‐repair responses. Conversely, downregulated DEGs in the Cu‑ZIF8‑Ru vs Cu^2^^+^ comparison showed significant enrichment for pathways involved in flagellar assembly and motility (Figure S28C), ABC transporters and transmembrane transport, as well as metal‐ion response/efflux systems and the tricarboxylic acid (TCA) cycle. This transcriptional repression collectively indicates that Cu‑ZIF8‑Ru suppresses bacterial motility, compromises nutrient and metal ion transport, disables metal‑tolerance mechanisms, and impairs central energy metabolism. These broad inhibitory effects are attributable to the severe envelope damage and intracellular Cu^2^^+^ overload induced by the nanozyme, the latter of which directly disrupts TCA cycle function. In the Cu‑ZIF8‑Ru+Laser vs Cu^2^^+^ comparison, upregulated DEGs exhibited an extension of the compensatory metabolic profile, with more pronounced enrichment in ribosome‐related pathways, Fe─S cluster assembly, sulfur metabolism, and metal binding (Figure 7D). This pattern indicates that MDR *E. coli* was compelled to intensify biosynthetic and repair activities under greater oxidative duress. The downregulated DEGs further underscored a suppression of flagella‐driven motility, metal‐ion transmembrane transport, membrane components, and transmembrane transporter complexes (Figure S28D), pointing to more severe disruption of ion homeostasis and chemotaxis. Thus, laser irradiation amplified the ROS stress induced by Cu‑ZIF8‑Ru, exacerbating defects in motility and transmembrane integrity, which accounts for the observed enhancement in bactericidal and anti‑biofilm efficacy.

To delineate the specific metabolic pathways involved, we performed KEGG enrichment analysis on the DEGs from the Cu‐ZIF8‐Ru vs Cu^2^^+^ and Cu‐ZIF8‐Ru+Laser vs Cu^2^^+^ comparisons (Figure S29A,B). For the Cu‐ZIF8‐Ru vs Cu^2^^+^ comparison, upregulated DEGs were significantly enriched in pathways including sulfur metabolism, amino acid and lipid biosynthesis, ribosome/RNA polymerase functions, peptidoglycan biosynthesis, and redox metabolism (Figure S29C). This coordinated upregulation reveals that, relative to free Cu^2^^+^, Cu‐ZIF8‐Ru imposes a compounded metal‐ion and oxidative stress, inflicts broader envelope damage, and thereby activates extensive biosynthetic and repair programs to maintain core functions such as translation, cell wall integrity, and redox balance. Notably, the upregulation of pyruvate and glutathione metabolism indicates aggravated oxidative stress and incipient bioenergetic failure. Conversely, the downregulated DEGs were significantly enriched in pathways governing flagellar assembly, quorum sensing, ABC transporters, the tricarboxylic acid (TCA) cycle, and *E. coli* biofilm formation (Figure 7E). This suppression profile demonstrates that Cu‐ZIF8‐Ru concurrently represses bacterial motility, disrupts nutrient/metal transport and efflux systems, and impairs central energy metabolism. These transcriptional changes provide a coherent molecular explanation for our phenotypic observations of membrane disruption, ATP depletion, Cu^2^^+^ overload, and biofilm inhibition. Under laser activation, this already heightened stress response was further amplified. The upregulated biosynthetic and repair pathways were intensified, with particularly strong enhancement in sulfur compound biosynthesis and amino acid metabolism (Figure S29D). This implies even more severe damage to Fe─S cluster‐containing enzymes and the electron transport chain, accounting for the superior efficacy of the photoactivated treatment. The downregulated DEGs in the Cu‐ZIF8‐Ru+Laser vs Cu^2^^+^ comparison showed broader suppression of flagellar assembly, ABC transporters, two‐component systems, quorum sensing, biofilm formation, and the TCA cycle, alongside impairments in multiple carbon and nucleotide metabolism pathways (Figure 7F). This indicates that photoactivation exacerbates the disruption of transmembrane homeostasis and energy metabolism. The KEGG results systematically show that Cu‐ZIF8‐Ru upregulates metabolic compensation and repair pathways while downregulating those essential for transport, energy production (TCA cycle), and community behaviors. This disruption is intensified by laser irradiation, confirming that the nanozyme's bactericidal effect stems from the exhaustion of bioenergetic functions.

To functionally validate the proposed mechanisms, we examined representative gene sets across treatments in MDR *E. coli*. A heatmap of key gene clusters, organized by functional category, is presented in Figure 7G (orange: upregulated; blue: downregulated). Compared to the Control and free Cu^2^^+^ groups, Cu‐ZIF8‐Ru treatment significantly upregulated Cu^2^^+^ efflux genes (copA, cueO, cusC, cusA), confirming intracellular Cu^2^^+^ overload. Concurrently, the downregulation of Zn^2^^+^ uptake genes (znuA, znuB) and upregulation of Zn^2^^+^ efflux genes (zntA, zntT) suggested Zn^2^^+^ overload, consistent with TEM‐EDS findings. Genes involved in cell‐wall/membrane biogenesis and permeability were upregulated, indicating active barrier disruption and remodeling. Furthermore, markers of oxidative stress, antioxidant response, DNA damage, and lipid peroxidation (LPO) were broadly elevated, providing direct transcriptional evidence for ROS accumulation and membrane lipid damage. Nutrient transporters and electron‐transport‐chain genes were broadly downregulated, and multiple TCA‐cycle enzymes showed decreased expression, revealing energy inhibition and confirming a starvation state. Notably, repair pathways including Fe─S cluster biogenesis, sulfur‐/lipoic‐acid metabolism, and protein lipoylation were compensatorily upregulated, consistent with preferential damage by Cu^2^^+^ overload plus ROS to Fe─S enzyme systems and the respiratory chain. Upon laser irradiation, these trends intensified: stress/repair responses increased further, whereas transport and energy pathways were more severely suppressed. This presents a typical pattern in which Cu^2^^+^ overload synergizes with amplified ROS to unbalance energy supply/demand and exacerbate oxidative injury, thereby enhancing bacteriostatic and bactericidal effects. Cu‐ZIF8‐Ru also suppressed TCA‐related enzymes and markedly enhanced lipid‐peroxidation signature pathways. To biochemically validate these findings, we further measured the activity of the pyruvate dehydrogenase complex (PDH) and the level of malondialdehyde (MDA), a lipid‐peroxidation product. PDH activity decreased sequentially from the Control to the Cu^2^^+^, Cu‐ZIF8‐Ru, and Cu‐ZIF8‐Ru+Laser groups, reaching its lowest point under laser irradiation (Figure 7H). This trend agreed with GO/KEGG observations of suppressed TCA cycle/electron transport chain, membrane‐potential depolarization, and ATP reduction, indicating that Cu‐ZIF8‐Ru effectively blocks the TCA cycle, with laser irradiation further amplifying this inhibitory effect. MDA levels were significantly elevated by Cu‐ZIF8‐Ru relative to free Cu^2^^+^ and the Control, and were maximized upon laser irradiation (Figure 7I), demonstrating a marked induction of lipid peroxidation in MDR *E. coli* by the Cu‐ZIF8‐Ru nanozyme. In summary, Cu‐ZIF8‐Ru exerts a multifaceted attack on MDR *E. coli*: it disrupts the bacteria envelope, induces intense lipid peroxidation, and blocks the TCA cycle and respiratory chain. Collectively, this multifaceted attack mirrors key hallmarks of copper‐induced cell death (cuproptosis) in eukaryotes. These findings strongly suggest that Cu‐ZIF8‐Ru induces a cuproptosis‐like bacterial death pathway in multidrug‐resistant bacteria.

### 2.8 In Vivo Therapeutic Effect of Cu‐ZIF8‐Ru Against Acute MDR‐UTIs

**FIGURE 8:**
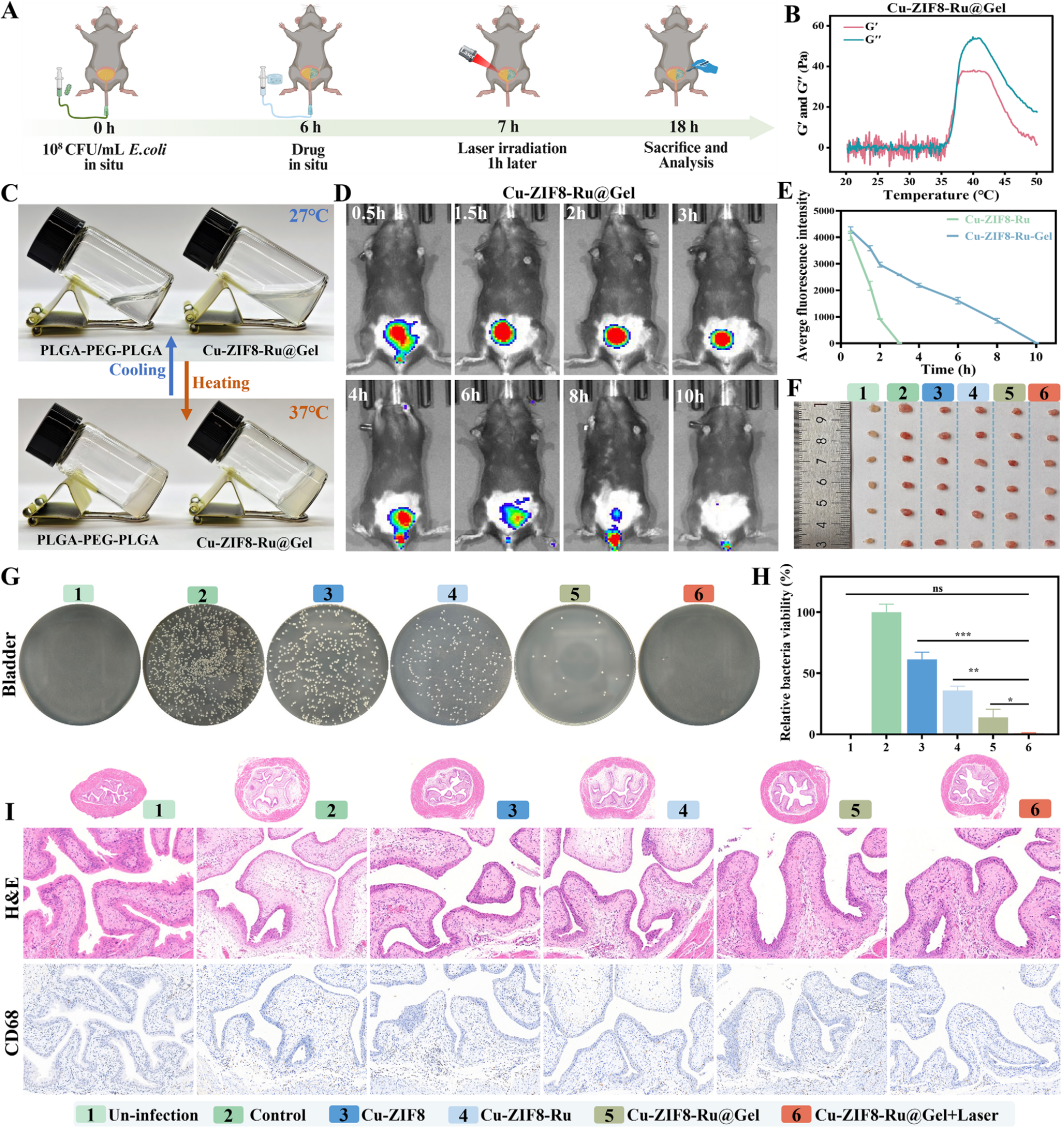
Therapeutic efficacy of Cu‐ZIF8‐Ru@Gel in acute MDR‐UTIs. (A) Therapeutic schedule for establishing the acute MDR‐UTIs model. (created with BioRender.com) (B) Rheological analysis demonstrating the temperature‐dependent sol‐gel transition of Cu‐ZIF8‐Ru@Gel. (C) Photographs demonstrating the reversible sol‐gel transformation of Cu‐ZIF8‐Ru@Gel at 27°C (sol) and 37°C (gel). (D) In vivo bioluminescence imaging of mice at different time points after intravesical instillation of ICG‐labeled Cu‐ZIF8‐Ru@Gel. (E) Fluorescence decay curves comparing Cu‐ZIF8‐Ru and Cu‐ZIF8‐Ru@Gel retention in the bladder. (F) Photographs of isolated bladders from different treatment groups. (G) Colony formation assay of bladder tissues showing bacterial survival in each group. (H) Quantitative analysis of relative bacterial viability in bladder tissues (n = 6). (I) H&E and CD68 staining of bladder tissues from different treatment groups.

Given the demonstrated biosafety and excellent bactericidal activity of Cu‐ZIF8‐Ru against MDR‐bacteria, we further investigated its therapeutic potential in MDR‐UTIs. Acute UTIs are characterized by a sudden onset and short duration, primarily caused by pathogenic bacteria colonizing the urinary tract. We successfully established an acute UTIs model by intravesical instillation of MDR *E. coli* for 6 h (Figure S30), which induced early pathological features, including bladder urothelial edema and inflammatory infiltration without widespread tissue necrosis. The complete workflow for the acute UTIs animal study is shown in Figure 8A. MDR‐UTIs are exceptionally difficult to manage due to multi‐antibiotic resistance and periodic urine dilution that reduces drug efficacy. To address this challenge, we leveraged the inherent adhesive property of Cu‐ZIF8‐Ru toward MDR bacteria to improve local retention. Furthermore, we incorporated Cu‐ZIF8‐Ru into the thermosensitive, biodegradable PLGA‐PEG‐PLGA hydrogel (Cu‐ZIF8‐Ru@Gel). This formulation transitions from an injectable solution to a stable gel at body temperature, thereby resisting urine washout and enabling sustained release of the nanozyme within the mildly acidic urinary microenvironment. At the physiologically relevant temperature of ∼37 °C, rheological studies verified the sol‐gel transition of PLGA‐PEG‐PLGA and Cu‐ZIF8‐Ru@Gel (Figure 8B). The ability of Cu‐ZIF8‐Ru@Gel to undergo this transition at body temperature supports its feasibility for clinical applications. Moreover, the incorporation of Cu‐ZIF8‐Ru was found to preserve the thermoresponsive gelation while resulting in a denser gel matrix with increased viscosity (Figure S31). The phase‐transition profile of Cu‐ZIF8‐Ru@Gel (Figure 8C) confirms its suitability for in situ gelation in the bladder, exhibiting good injectability. We then evaluated the stability and release kinetics of Cu‐ZIF8‐Ru@Gel in PBS and artificial urine. The gel maintained its morphology in PBS over 30 h but underwent marked volume shrinkage in artificial urine (Figure S32A,B), indicating urine‐promoted erosion. Correspondingly, the cumulative release of Cu‐ZIF8‐Ru in artificial urine reached approximately 90% within 30 h (Figure S32C), demonstrating sustained release in the urine‐like environment. Finally, in vivo fluorescence imaging after intravesical instillation of ICG‐labeled Cu‐ZIF8‐Ru showed that Cu‐ZIF8‐Ru@Gel provided significantly prolonged bladder residence (Figure 8D), with fluorescence persisting for up to 10 h. In comparison, the nanozyme alone was largely cleared within 3 h (Figure S33). Quantitative analysis of the fluorescence decay further confirmed the enhanced retention capability of the hydrogel formulation (Figure 8E). These findings collectively support Cu‐ZIF8‐Ru@Gel as a promising therapy for MDR‐UTIs.

Following the treatment regimen (Figure 8F), control bladders exhibited pronounced hyperemia and edema. These pathological signs were markedly reduced in the Cu‐ZIF8‐Ru@Gel group relative to the Cu‐ZIF8 and Cu‐ZIF8‐Ru groups. Most notably, the gross appearance of bladders in the Cu‐ZIF8‐Ru@Gel+Laser group closely resembled that of healthy, uninfected controls. Quantitative culture of bladder homogenates revealed dense bacterial colonies in the control group (Figure 8G), confirming a high bacterial burden. A comparative reduction in bacterial colonies was observed in the Cu‐ZIF8‐Ru group relative to Cu‐ZIF8, indicative of enhanced, albeit not optimal, antibacterial activity. A further significant decrease in bacterial load was achieved with the Cu‐ZIF8‐Ru@Gel formulation, attributable to the hydrogel‐mediated enhancement of retention and therapeutic efficacy. Near‐complete bacterial eradication was attained only with the inclusion of laser irradiation in the Cu‐ZIF8‐Ru@Gel+Laser group. These observations were quantitatively confirmed by CFU enumeration, which established the significant in vivo inhibitory activity of the Cu‐ZIF8‐Ru@Gel complex against MDR *E. coli* (Figure 8H). Histopathological evaluation by H&E staining further delineated the therapeutic outcomes (Figure 8I). Compared to uninfected controls, bladders in the infection control group exhibited features characteristic of acute cystitis, marked by epithelial edema, blunted mucosal rugae, and diffuse neutrophil infiltration within the lamina propria. Cu‐ZIF8 treatment initiated epithelial recovery, attenuating hemorrhage and edema. The Cu‐ZIF8‐Ru group demonstrated further architectural restoration, with inflammation resolving to only focal neutrophil infiltration. Pathological morphology in the Cu‐ZIF8‐Ru@Gel group closely approached the physiological state, exhibiting merely mild residual inflammation. The most pronounced recovery was observed in the Cu‐ZIF8‐Ru@Gel+Laser group, which displayed intact mucosa and crisp rugae, most closely resembling the uninfected tissue architecture. Inflammatory activity was assessed via immunohistochemical staining for the macrophage marker CD68. Diffuse CD68 positivity alongside marked macrophage aggregation in the lamina propria was observed in the control group. A graded reduction in macrophage infiltration was noted across treatment groups: marginal in the Cu‐ZIF8 group, substantial (with only focal positivity) in the Cu‐ZIF8‐Ru group, and pronounced (to near‐normal levels) in the Cu‐ZIF8‐Ru@Gel group. Near‐complete suppression was evident in the Cu‐ZIF8‐Ru@Gel+Laser group, which displayed minimal CD68 expression and macrophage infiltration, indicating potent anti‐inflammatory efficacy. Histopathological examination of the kidney (Figure S34) and other major organs (heart, liver, spleen, lung) (Figure S35) revealed no evidence of treatment‐related pathological injury. Histological analysis revealed intact renal glomeruli and tubules across all treatment groups, with no significant pathology beyond mild congestion in infection controls. These observations confirm the good biocompatibility of the intravesical Cu‐ZIF8‐Ru@Gel system. Collectively, the data demonstrate that Cu‐ZIF8‐Ru@Gel achieves excellent biocompatibility, enhances therapeutic efficacy by combining prolonged intravesical residence with efficient drug delivery, and potently inhibits and kills MDR *E. coli*, fulfilling the core requirements for an effective MDR‐UTIs therapy.

### 2.9 In Vivo Therapeutic Effect of Cu‐ZIF8‐Ru Against Recurrent MDR‐UTIs

**FIGURE 9:**
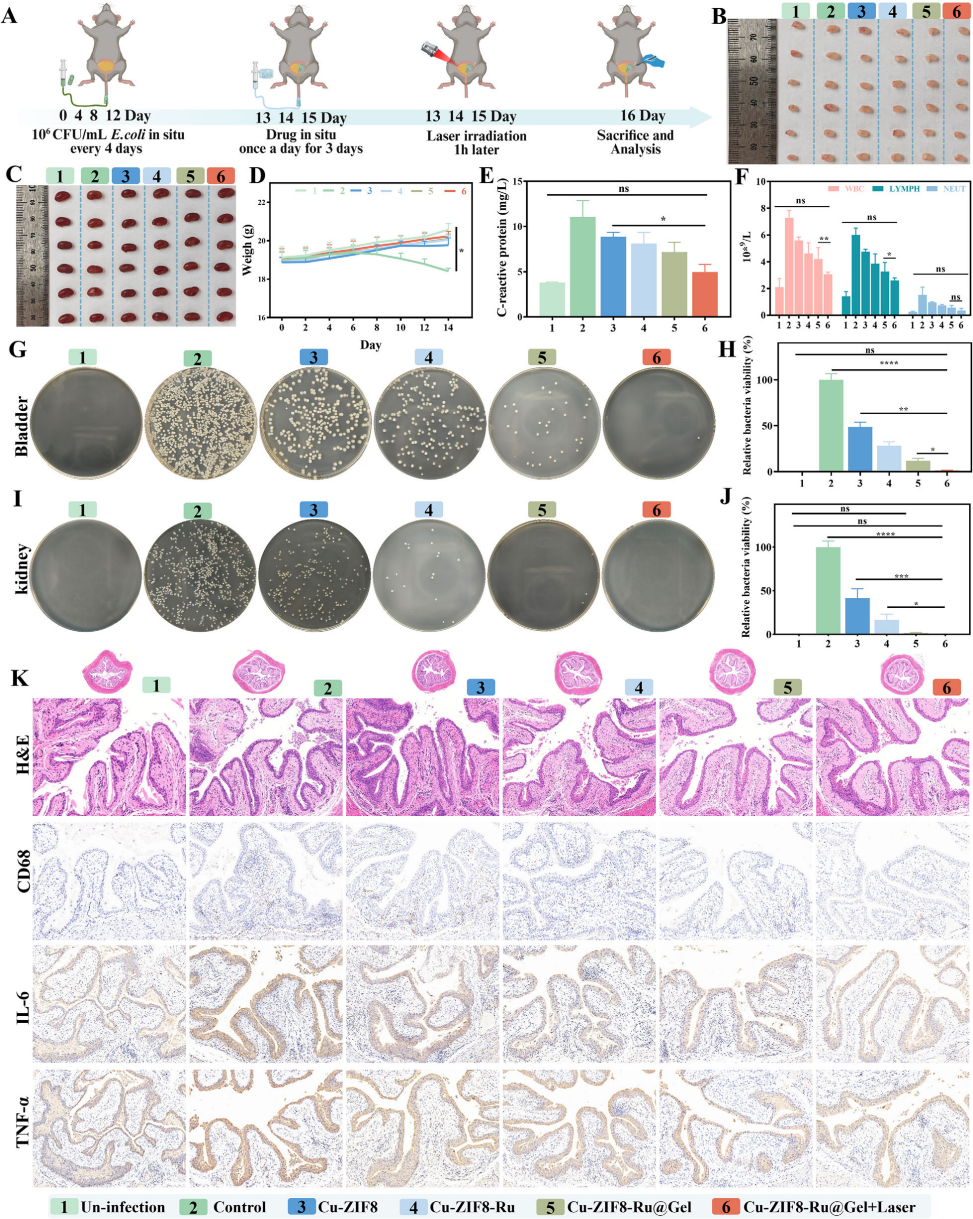
Therapeutic efficacy of Cu‐ZIF8‐Ru@Gel in recurrent MDR‐UTIs. (A) Therapeutic schedule for establishing the recurrent MDR‐UTIs. (created with BioRender.com) (B) Representative images of excised bladders from different groups. (C) Representative images of the excised kidney from different groups. (D) Body weight changes of mice during treatment (n = 6). (E) Serum C‐reactive protein levels in different groups (n = 3). (F) Peripheral blood cell analysis, including WBC, lymphocytes, and neutrophils (n = 3). (G) Colony formation assay of bladder tissues showing bacterial survival. (H) Quantitative analysis of relative bacterial viability in bladder tissues (n = 6). (I) Colony formation assay of kidney tissues (n = 6). (J) Quantitative analysis of relative bacterial viability in kidney tissues (n = 6). (K) H&E staining and immunohistochemical analysis (CD68, IL‐6, TNF‐α) of bladder tissues.

Given the potent antibacterial activity of Cu‐ZIF8‐Ru@Gel observed in acute MDR‐UTIs, we hypothesized its utility in the more complex setting of the recurrent MDR‐UTIs. A recurrent MDR‐UTIs model was established by intravesical instillation of MDR *E. coli* three times per week at 4‐day intervals (Figure S36). Histopathological assessment of bladder tissue confirmed successful model induction, revealing marked urothelial thickening, hyperplastic rugae, loss of superficial umbrella cell continuity, and diffuse neutrophil infiltration. The full experimental workflow for the recurrent MDR‐UTI model is illustrated in Figure 9A. Macroscopic assessment of bladder tissues revealed that, compared to the uninfected group, the infection control group displayed increased bladder size and patchy hyperemia, consistent with severe inflammation (Figure 9B). Bladder distension was notably reduced in the Cu‐ZIF8‐Ru@Gel group relative to the Cu‐ZIF8 and Cu‐ZIF8‐Ru groups. The Cu‐ZIF8‐Ru@Gel+Laser group most closely approximated the uninfected controls in both organ size and coloration, indicating a substantial mitigation of inflammatory pathology. Consistent with these observations, renal analysis revealed that the infection control group presented with reduced kidney size and pallor, indicative of ascending infection (Figure 9C). The renal gross morphology in the Cu‐ZIF8‐Ru@Gel group more closely approximated the uninfected state than did the Cu‐ZIF8 and Cu‐ZIF8‐Ru groups, with the Cu‐ZIF8‐Ru@Gel+Laser group exhibiting the most complete restoration of normal appearance. Variations in murine body weight provided an indirect assessment of overall disease severity (Figure 9D). At the endpoint, the control group exhibited significant weight loss. In contrast, the body weight of mice in the Cu‐ZIF8‐Ru@Gel and Cu‐ZIF8‐Ru@Gel+Laser groups was comparable to that of the uninfected group, indicating effective mitigation of disease progression. Systemic inflammation was further evaluated by measuring serum C‐reactive protein (CRP) levels and performing complete blood counts. As shown in Figure 9E, the control group exhibited markedly elevated serum CRP levels relative to the uninfected group, indicating severe systemic inflammation. CRP was moderately reduced in the Cu‐ZIF8‐Ru@Gel group and most closely approximated baseline in the Cu‐ZIF8‐Ru@Gel+Laser group, demonstrating potent anti‐inflammatory activity. Correspondingly, white blood cell (WBC), neutrophil, and lymphocyte counts were significantly elevated in the control group (Figure 9F), consistent with an active infection. These hematological parameters showed a progressive decline in the Cu‐ZIF8‐Ru@Gel group and were nearly normalized with laser adjunct therapy. Collectively, these results demonstrate that Cu‐ZIF8‐Ru@Gel potently controls inflammation at both the local and systemic levels in recurrent MDR‐UTIs, with laser irradiation significantly enhancing this therapeutic effect.

Following treatment, bacterial burdens in the bladder and kidney were quantified by homogenizing and plating. As shown in Figure 9G, dense bacterial colonies were observed in the control group, indicating a high bladder bacterial load. The Cu‐ZIF8‐Ru group produced fewer colonies than the Cu‐ZIF8 group, suggesting improved yet incomplete control of the intravesical infection. Antibacterial efficacy was substantially enhanced in the Cu‐ZIF8‐Ru@Gel group, where only scattered colonies were detected. Notably, nearly no colonies were detectable in the Cu‐ZIF8‐Ru@Gel+Laser group, confirming the strongest bactericidal effect upon laser activation. These qualitative observations were corroborated by quantitative colony‐forming unit (CFU) counts from bladder homogenates (Figure 9H). Renal cultures from the control group yielded abundant bacterial colonies (Figure 9I), confirming ascending infection. In contrast, colony counts in both the Cu‐ZIF8‐Ru@Gel and Cu‐ZIF8‐Ru@Gel+Laser groups were nearly undetectable compared to the Cu‐ZIF8 and Cu‐ZIF8‐Ru groups. This demonstrates that effective eradication of bladder infection prevented renal colonization by MDR *E. coli*, a conclusion further supported by quantitative kidney CFU counts (Figure 9J). Histopathological evaluation of bladder and kidney tissues was performed to further determine therapeutic efficacy against recurrent MDR‐UTIs. The control bladder tissues revealed hallmark pathological features, including papillary hyperplasia, urothelial edema, and dense inflammatory infiltrates (Figure 9K). In comparison, the Cu‐ZIF8 group exhibited persistent diffuse neutrophilic infiltration. A transition toward recovery was evident in the Cu‐ZIF8‐Ru group, characterized by architectural regularization and diminished neutrophil counts. This recovery was more pronounced in the Cu‐ZIF8‐Ru@Gel group, where mucosal morphology neared normal, and inflammation was markedly suppressed. The most significant restoration was observed in the Cu‐ZIF8‐Ru@Gel+Laser group, which presented minimal neutrophilic infiltrate and an architecture most closely resembling the uninfected controls. Assessment of macrophage infiltration via CD68 immunohistochemistry revealed extensive positivity in control tissues, indicative of persistent inflammation. A sequential reduction in CD68 immunoreactivity was observed: modest in Cu‐ZIF8‐Ru versus Cu‐ZIF8, substantial in Cu‐ZIF8‐Ru@Gel, and approaching control levels in the Cu‐ZIF8‐Ru@Gel+Laser group. Parallel evaluation of the pro‐inflammatory cytokine IL‐6 showed intense staining in controls, which remained strong with Cu‐ZIF8, was moderately reduced with Cu‐ZIF8‐Ru, and was markedly suppressed with Cu‐ZIF8‐Ru@Gel. Minimal IL‐6 expression was detected in the Cu‐ZIF8‐Ru@Gel+Laser group, confirming the most potent anti‐inflammatory activity and effective mitigation of the cytokine storm. TNF‐α immunostaining revealed widespread expression in control tissues, consistent with sustained inflammation. A modest reduction was observed with Cu‐ZIF8‐Ru, followed by predominantly faint positivity with Cu‐ZIF8‐Ru@Gel. Near‐abolition of the TNF‐α signal was specific to the Cu‐ZIF8‐Ru@Gel+Laser group. These findings indicate that Cu‐ZIF8‐Ru@Gel comprehensively addresses recurrent MDR‐UTI pathology by alleviating tissue edema and damage, inhibiting macrophage infiltration, and downregulating pivotal inflammatory mediators (IL‐6, TNF‐α), culminating in effective infection clearance and inflammation suppression.

Given the recognized potential for recurrent MDR‐UTIs to ascend and cause renal complications, histopathological assessment of kidney tissues was essential. Compared to the uninfected group, H&E‐stained kidney sections from the control group exhibited moderate neutrophilic infiltration, vascular congestion, and mild degeneration of the renal tubular epithelium (Figure S37). These pathological alterations were partially mitigated in the Cu‐ZIF8 and Cu‐ZIF8‐Ru groups. In contrast, the renal architecture in both the Cu‐ZIF8‐Ru@Gel and Cu‐ZIF8‐Ru@Gel+Laser groups closely resembled that of uninfected kidneys, demonstrating pronounced suppression of inflammation and edema, with no indications of treatment‐related necrosis or hemorrhage. CD68 immunostaining further demonstrated a diffuse infiltration of CD68‐positive macrophages in the renal tissues of the control group, indicative of active recruitment. This signal was only marginally attenuated in the Cu‐ZIF8 and Cu‐ZIF8‐Ru groups. In contrast, a marked reduction in CD68 positivity was observed in the Cu‐ZIF8‐Ru@Gel group. CD68 expression in the Cu‐ZIF8‐Ru@Gel+Laser group returned to near‐baseline levels, confirming that the laser‐augmented formulation effectively suppressed the ascending renal infection. A corresponding trend was observed for the pro‐inflammatory cytokines IL‐6 and TNF‐α. The control group demonstrated mild immunopositivity, consistent with local cytokine release. Expression of both IL‐6 and TNF‐α was markedly decreased in the Cu‐ZIF8 and Cu‐ZIF8‐Ru groups, and was minimal to nearly absent in the Cu‐ZIF8‐Ru@Gel and Cu‐ZIF8‐Ru@Gel+Laser groups. This confirms effective control of the ascending renal infection. Overall, the renal histological architecture in the Cu‐ZIF8‐Ru@Gel and Cu‐ZIF8‐Ru@Gel+Laser groups closely resembled that of uninfected controls, with significant downregulation of CD68, IL‐6, and TNF‐α, demonstrating effective suppression of ascending infection in the absence of detectable renal toxicity. Assessment of systemic hematological parameters (RBCs, hemoglobin, platelets) revealed values in all treatment groups that were comparable to those in uninfected controls (Figure S38), demonstrating no detectable toxicity to the hematopoietic system. Consistent with this, histopathological evaluation of vital organs (heart, liver, spleen, lung) following repeated administration showed preserved architecture with no notable lesions (Figure S39). These findings collectively substantiate the good biocompatibility and biosafety of the Cu‐ZIF8‐Ru@Gel formulation.

## 3 Conclusions

This study addresses the critical clinical challenges in managing MDR‐UTIs, particularly the scarcity of effective drugs and the periodic dilution of therapeutics due to urine clearance. To overcome these barriers, we developed a Cu‐ZIF8‐Ru nanozyme and encapsulated it within a thermosensitive hydrogel. This formulation enables intravesical in situ gelation and sustained release, thus enhancing the suppression and eradication of MDR‐bacterial populations and achieving effective MDR‐UTIs therapy. The key findings of this study are summarized below: (1) The ruthenium complex [Ru(phen)_2_dppz]^2^^+^ was integrated with Cu‐ZIF8 via atomic deposition to construct a Cu‐ZIF8‐Ru nanozyme featuring an asymmetric heterobimetallic Cu─N─Ru active site. XAFS and DFT analyses verified that this unique structure promotes interfacial electron transfer, thereby collectively enhancing the OXD‐, POD‐, GSH‐Px‐like nanozyme activities. (2) The Cu‐ZIF8‐Ru nanozyme degrades in the mildly acidic urinary environment, efficiently releasing Cu^2^^+^, Zn^2^^+^, and Ru(II). Upon laser irradiation, it amplifies ROS‐induced damage to the bacterial envelope, thereby increasing membrane permeability and accelerating Cu^2^^+^ accumulation. This cascade intensifies intracellular oxidative stress and enhances the overall bactericidal effect. More importantly, the Cu‐ZIF8‐Ru nanozyme maintains a robust capacity to inhibit and disrupt biofilms formed by multidrug‐resistant bacteria, even under the challenging conditions of protein‐rich, high‐glucose, and low‐pH environments. The observed bactericidal and bacteriostatic effects are primarily attributed to its efficient adhesion to the bacterial surface and the acid‐triggered release of Cu^2^^+^ and Ru(II). This combined action elevates the local concentration of active agents at the bacterial periphery, thereby significantly augmenting antimicrobial efficacy. (3) RNA‐seq analysis demonstrated that Cu‐ZIF8‐Ru inflicts multi‐faceted damage on MDR *E. coli*, including cell envelope compromise, elevated lipid peroxidation, and severe disruption of the respiratory electron transport chain. Intracellular Cu^2^^+^ overload further perturbed the TCA cycle and diminished PDH activity. These perturbations triggered a widespread compensatory response, activating the compensatory upregulation of pathways cell envelope biogenesis, ribosome function, Fe─S cluster assembly, sulfur acid metabolism, metal‐ion binding, antioxidant defense, and DNA damage repair. In contrast, a coordinated downregulation was observed in nutrient import and energy metabolism pathways, such as ABC transporters, the respiratory chain, and the TCA cycle. This pattern reflects a synergistic effect between Cu^2^^+^ overaccumulation and ROS, which disrupts cellular energy homeostasis and exacerbates oxidative injury, indicating that Cu‐ZIF8‐Ru induces a cuproptosis‐like process in bacteria. (4) The Cu‐ZIF8‐Ru nanozyme demonstrated negligible cytotoxicity and hemolytic activity in vitro, confirming its favorable biocompatibility. After encapsulation in a PLGA‐PEG‐PLGA thermoresponsive hydrogel, the formulation enabled intravesical instillation with in situ gelation, providing bladder retention and stable sustained release. thereby yielding superior antibacterial efficacy over the free Cu‐ZIF8‐Ru nanozyme. The Cu‐ZIF8‐Ru nanozyme platform demonstrated robust efficacy in preclinical models of acute and recurrent MDR‐UTIs, simultaneously eliminating resistant bacteria, controlling inflammation, and blocking ascending infection. Overall, this work establishes Cu‐ZIF8‐Ru as an efficient, controllable, and low‐toxicity intravesical antimicrobial strategy that addresses urgent clinical needs in MDR‐UTIs management and demonstrates strong translational promise.

## Author Contributions

L.G.L., L.H.X., and W.W.Q. conceived the idea and designed the project. L.G.L. performed the experiments and analyzed the results. L.G.L., Z.S.K., L.J.G., and L.W. assisted with the experiment design and data analyses. L.G.L. wrote and revised the original draft of the manuscript and performed XANES, EXAFS, and the DFT simulations. L.H.X. and W.W.Q. reviewed and edited the manuscript. L.H.X. and W.W.Q. supervised the whole project. All authors discussed the results and commented on the manuscript.

## Conflicts of Interest

The authors declare no conflicts of interest.

## Supporting information




**Supporting File**: advs74091‐sup‐0001‐SuppMat.docx.

## Data Availability

The data that support the findings of this study are available in the supplementary material of this article.
