# Supplementary material for: Asymmetric Cu─N─Ru Bridgedsite Nanozyme‐Loaded Injectable Thermogel Boosts Cuproptosis‐Like Death for Multidrug‐Resistant Urinary Tract Infections
Source: Adv Sci (Weinh). 2026 Jan 28;13(19):e20705. doi: 10.1002/advs.202520705 (PMC13045214; doi:10.1002/advs.202520705)
Supplement: Supplementary file 1 — Supporting File: advs74091‐sup‐0001‐SuppMat.docx. [file ADVS-13-e20705-s001.docx]

**Asymmetric Cu-N-Ru Bridgedsite Nanozyme-loaded Injectable Thermogel boosts Cuproptosis-like Death for Multidrug-Resistant Urinary Tract Infections**

Guanlin Li^1#^, Shike Zhang^1#^, Jinggong Liu^4#^, Xin Wang^2^, Heng Wang^2^, Hongcan Yang^1^, Yuhao Zhou^1^, Wen Liu^3*^, Hongxing Liu^2*^, Wenqi Wu^1*^

Guanlin Li, Shike Zhang, Hongcan Yang, Yuhao Zhou, Wenqi Wu

[1] Department of Urology, the Second Affiliated Hospital of Guangzhou Medical University, Guangzhou, China.

Xin Wang, Heng Wang, Hongxing Liu

[2] Department of Urology, The First Affiliated Hospital, Guangdong Provincial Key Laboratory of Urological Diseases, Guangdong Engineering Research Center of Urinary Minimally Invasive Surgery, Robot and Intelligent Equipment, Guangzhou Institute of Urology, Guangzhou Medical University, Guangzhou, China.

Wen Liu

[3] School of Public Health, Guangzhou Medical University, Guangzhou 511436, China.

Jinggong Liu

[4] State Key Laboratory of Traditional Chinese Medicine Syndrome, Orthopedics Department, The Second Affiliated Hospital of Guangzhou University of Chinese Medicine, Guangzhou 510120, China.

Correspondence E-mail: [**liuwen@gzhmu.edu.cn**](mailto:liuwen@gzhmu.edu.cn) (Wen Liu), [**liuhongxing@gzhmu.edu.cn**](mailto:liuhongxing@gzhmu.edu.cn) (Hongxing Liu), [**2009681012@gzhmu.edu.cn**](mailto:2009681012@gzhmu.edu.cn) (Wenqi Wu).

\# These authors contributed equally to this work.

**Experimental Section**

**Materials and Characterization.**

**Materials.** All reagents were analytical grade and used without further purification. Cu(NO₃)₂, Zn(NO₃)₂, 2-methylimidazole, 3,3′,5,5′-tetramethylbenzidine (TMB), *o*-phenylenediamine (OPD), and 5,5′-dithiobis(2-nitrobenzoic acid) (DTNB) were purchased from Macklin. ipyridophenazine (dppz), 1,10-phenanthroline, lithium chloride, N,N-dimethylformamide, acetone, potassium hexafluorophosphate (KPF₆), acetonitrile (CH₃CN), diethyl ether, and indocyanine green (ICG) were obtained from Sigma-Aldrich. A 2.5% glutaraldehyde solution and methyl violet were purchased from Aladdin. The ROS fluorescent probe 2′,7′-dichlorofluorescein diacetate (DCFH-DA) was supplied by Solarbio. Hydrochloric acid, methanol, absolute ethanol, and Triton X-100 solution were obtained from Guangzhou Chemical Reagent Factory. The membrane potential probe DiSC₃(5) was purchased from Guangzhou Meilun Biotechnology. The CCK-8 kit, SYTO-9/PI Live/Dead Bacterial Viability Kit, malondialdehyde (MDA) assay kit, and ATP content assay kit were provided by Beyotime. The pyruvate dehydrogenase (PDH) activity assay kit was purchased from Boxbio Biotechnology. The BCA protein assay kit was obtained from Thermo. The Alamar Blue Cell Viability Assay Kit was purchased from Guangzhou Yongjin Biotechnology Co., Ltd. LB broth medium and LB agar plates were obtained from Jiangmen Kailin Trading. Fetal bovine serum (FBS), trypsin, DMEM medium, RPMI-1640 medium, and phosphate-buffered saline (PBS) were supplied by Guangzhou Weining Biotechnology.

**Synthesis of Cu-ZIF8.** Cu(NO₃)₂, Zn(NO₃)₂, 2-methylimidazole were dissolved in methanol under N₂. The solutions were mixed at room temperature and gently stirred for 4 h, followed by high-speed centrifugation. The precipitate was washed with methanol to yield Cu-ZIF8 MOFs with varied Zn:Cu ratios.

**Synthesis of ruthenium complexes [Ru(phen)₂dppz]²⁺**. Phenanthroline (0.6845 g), RuCl₃·3H₂O (0.50 g), and LiCl (0.80 g) were added to a 100 mL round-bottom flask with dimethylformamide (DMF, 20 mL) and stirred at 120 °C overnight. DMF was removed under reduced pressure, and the residue was treated with acetone (50 mL) and filtered to afford a brown-black solid, which was washed with ice water (10 mL) and acetone (50 mL) to give cis-[Ru(phen)₂Cl₂]. cis-[Ru(phen)₂Cl₂] (0.653 g) and dppz (1.10 g) were dissolved in EtOH/H₂O (50/25 mL) and refluxed under N₂ for 8 h to afford an orange product. The crude was purified by silica-gel chromatography (CH₃CN:toluene = 1:1), followed by anion exchange with KPF₆ (1.0 g). After removal of volatiles, the remaining solid was recrystallized from CH₃CN/Et₂O to obtain Ru(phen)₂dppz₂ (abbreviated as Ru(II)).[1]

**Synthesis of Cu-ZIF8-Ru nanozyme.** Cu-ZIF8 and a ruthenium complex were co-dissolved in DMSO at a 3:1 (w/w) mass ratio, the mixture was stirred at a low speed, while being heated under a nitrogen atmosphere and in a dark environment for 12 h. The solids were isolated by high-speed centrifugation and washed with methanol to obtain the Cu-ZIF8-Ru nanozyme.

**Synthesis of Cu-ZIF8-Ru@Gel.** PLGA-PEG-PLGA was synthesized under N₂ via ROP of D,L-lactide (LA) and glycolide (GA), initiated by PEG and catalyzed by Sn(Oct)₂. The crude mixture was immediately poured into deionized water and stirred overnight under N₂. The material was heated in a water bath to remove residual monomers and water-soluble low-molecular-weight species, then lyophilized to yield the PLGA-PEG-PLGA hydrogel. Cu-ZIF8-Ru was combined with the hydrogel at a 1:2 (v/v) ratio and stirred gently at room temperature overnight to obtain Cu-ZIF8-Ru@Gel.

**Preparation of ICG-Cu-ZIF8-Ru and ICG-Cu-ZIF8-Ru@Gel.** (1) ICG (500 µL, 1 mg/mL) was combined with Cu-ZIF8-Ru (500 µL, 100 µg/mL) and stirred for 12 h in the dark. The solids were isolated by high-speed centrifugation and resuspended to yield ICG-Cu-ZIF8-Ru. (2) The ICG-Cu-ZIF8-Ru dispersion was blended with PLGA-PEG-PLGA hydrogel at 1:2 (v/v) and gently stirred overnight at room temperature to afford ICG-Cu-ZIF8-Ru@Gel.

**Characterization.** Morphology of Cu-ZIF8-Ru was examined by SEM (Hitachi SU8100) and TEM (JEOL JEM-2100F) with EDS. Hydrodynamic size distributions were assessed using a Malvern Zetasizer Nano ZS90. XPS (Thermo Scientific K-Alpha) was referenced to adventitious carbon (C 1s = 284.6 eV). Powder XRD patterns were collected on a Bruker D8. ESR spectra were recorded at room temperature on a Bruker A300 at X-band (3.5 GHz). N₂ adsorption–desorption isotherms at 77 K were acquired on a Micromeritics 3Flex after vacuum degassing at 100 °C at least 6 h; surface areas and pore parameters were derived by BET and BJH, respectively. FT-IR spectra (4000-400 cm⁻¹) were obtained on a Nicolet iS5, and UV-Vis spectra on the Shimadzu UV-3600. Bacterial fluorescence imaging employed an Olympus fluorescence microscope. A 1000 mW, 660 nm laser was used for bacterial and *in vivo* experiments. Whole-slide histology was scanned with PathScope (GEN DigiPath). Rheology of PLGA-PEG-PLGA and Cu-ZIF8-Ru-Gel (sol-gel behavior) was characterized on a TA HR10 rheometer.

X-ray absorption fine structure (XAFS) data were processed using the ATHENA module of the IFEFFIT software package. Least-square curve fitting was performed using the ARTEMIS module within the same package.[[2-3] The structural parameters extracted from the fitting include: coordination number (CN) for the absorber–backscatter pair, interatomic distance (R), Debye–Waller factor (σ²), and inner potential shift (ΔE₀). The amplitude reduction factor (*S₀²) was fixed at 0.83 for Cu and 0.87 for Ru, as derived from the respective metal foil references. The estimated accuracies of the fitted parameters were as follows: CN, ±20%; R, ±1%; σ², ±20%; and ΔE₀, ±20%. Fitting was conducted in k-space over the range of 3.0–11.0 Å⁻¹ for the Cu environment and 3.0–12.0 Å⁻¹ for Ru, and in R-space over 1.0–2.8 Å for both. For wavelet transform (WT) analysis, the χ(k) data exported from ATHENA were imported into Igor Pro 6.10A. The WT magnitudes were calculated using a Morlet wavelet with parameters κ = 4 and σ = 2. The k-ranges applied were 3.0–11.0 Å⁻¹ for the sample and 3.0–12.0 Å⁻¹ for the reference standards, with R-ranges of 0–4.0 Å in both cases.

All density functional theory (DFT) calculations were performed using the Vienna Ab initio Simulation Package (VASP), utilizing the Perdew-Burke-Ernzerhof (PBE) generalized gradient approximation (GGA).[4-5] The projected augmented wave (PAW) method was employed to describe the ionic cores, and the wavefunctions were expanded using a plane-wave basis set with a kinetic energy cutoff of 500 eV.[6-7] The Gaussian smearing method with a width of 0.05 eV was employed for partial orbital occupancies. The convergence criteria for electronic self-consistency and geometry optimization were set to 10⁻⁵ eV and 0.05 eV/Å, respectively. Dispersion interactions were accounted for using Grimme's DFT-D3 method.[8] The adsorption energy (Eads) was calculated using the equation: Eads = Ead/sub – Ead – Esub, where Ead/sub, Ead, and Esub denote the total energy of the adsorbate/substrate complex, the isolated adsorbate, and the pristine substrate, respectively.

The free energy G was computed according to the relation: G = E + ZPE - TS

The total Gibbs free energy includes the DFT-calculated energy (E), zero-point energy (ZPE), and the entropic term (TS). Additionally, the vibrational entropy (Svib) was determined using the harmonic normal mode approximation as follows:

Where kB is Boltzmann constant, ℎ is Planck constant, and T represents temperature. Vibrational frequency calculations were performed to obtain zero-point energy (ZPE) corrections.

**Assessment of** **Multiple Enzyme Activities of Cu-ZIF8-Ru.** Cu-ZIF8-Ru displays oxidase-like (OXD), peroxidase-like (POD), and glutathione peroxidase-like (GSH-Px) activities. First, the OXD- and POD-like activities of Cu-ZIF8-Ru were investigated and evaluated using TMB and OPD as probes. Six groups with different components were set up: Cu-ZIF8-Ru+OPD/TMB+H_2_O_2_, OPD/TMB+H_2_O_2_, Cu-ZIF8-Ru+OPD/TMB, OPD/TMB+H_2_O_2_, OPD/TMB. The mixtures were incubated in Constant temperature incubator for 10 min to prevent further non-enzymatic oxidation or degradation of the products; the reactions were then quenched by acid, and the solutions were analyzed on a microplate reader. Next, the sensitivity of Cu-ZIF8-Ru to H₂O₂ was verified by establishing an equidistant gradient of H₂O₂ concentrations (0.5, 0.4, 0.3, 0.2, 0.1, and 0 mM). The sensitivity of H₂O₂ (0.2 mM) to Cu-ZIF8-Ru was then assessed by preparing seven Cu-ZIF8-Ru concentrations (200, 150, 100, 75, 50, 25, and 0 µg/mL). In addition, the OXD-like property of Cu-ZIF8-Ru was determined using the same seven Cu-ZIF8-Ru concentrations (200, 150, 100, 75, 50, 25, and 0 µg/mL). All results were measured and recorded with a microplate spectrophotometer. For GSH-Px-like activity and GSH depletion, DTNB was used as the probe and measured on a microplate reader. Cu-ZIF8-Ru solutions at 50 and 100 µg/mL were prepared and supplemented with 1.0 mM GSH. Then 900 µL PBS was added to 100 µL of the above solution along with 10 µL DTNB (0.5 mM). Absorbance values were recorded and analyzed at different time points.

The Cu-ZIF8-Ru nanozyme was dispersed in 2 mL of a pH 5.5 solution and incubated for various durations (0, 2, 4, 6, 8, 12, 24 h). After incubation, the samples were centrifuged at 5000 rpm to collect the precipitate, which was then resuspended. Assay mixtures were prepared in three groups: TMB only, Cu-ZIF8-Ru + TMB, and Cu-ZIF8-Ru + TMB + H₂O₂. The nanozyme was diluted to 50 μg/mL with ultrapure water prior to use. The TMB probe was added to the respective solutions, and the mixtures were incubated in a constant-temperature incubator for 10 min. Subsequently, the reactions were quenched with acid to prevent further non-enzymatic oxidation or degradation of the product. All results were detected and recorded using a microplate reader.

**Biosafety assessment.** Cu-ZIF8-Ru samples with copper contents of 10%, 20%, and 30% were incubated with SV-HUC-1 and HUVEC cells (4 × 10⁴ cells/mL; 100 µL per well) in 96-well plates. Then, 100 µL of Cu-ZIF8-Ru with different copper contents (final concentration range 0-400 µg/mL) was added and incubated for 12 h. After adding 20 µL CCK-8 to each well and incubating for 2 h, cell viability was measured on a microplate spectrophotometer. Based on this screen, the Cu-ZIF8-Ru formulation with the safest concentration range was selected for further biosafety evaluation. Groups included: Ru(II), Cu-ZIF8, Cu-ZIF8-Ru, Ru(II)+Laser, Cu-ZIF8+Laser, and Cu-ZIF8-Ru+Laser. SV-HUC-1 cells were incubated with a fixed dose of Cu-ZIF8-Ru in 96-well plates for 5 h; cells in the laser groups were then exposed to a 660 nm laser (100 mW/cm²) for 10 min. After adding 20 µL CCK-8 and incubating for 2 h, cell viability was determined by microplate spectrophotometry. In addition, hemolysis assays using red blood cells from C57BL/6 mice were performed to assess the biosafety of Cu-ZIF8-Ru. Collected blood was centrifuged to isolate erythrocytes, which were resuspended in PBS and incubated with Cu-ZIF8-Ru (0-400 µg/mL) at 37 °C for 12 h. A 5 µL Triton X-100 solution was used to lyse erythrocytes as a positive control. After incubation, absorbance at 540 nm was recorded on a microplate reader to evaluate the impact of Cu-ZIF8-Ru on red blood cells.

***In vitro* antibacterial activity.**

**Screening of optimal antibacterial incubation conditions.**

The target strains used in this study were multidrug-resistant *Escherichia coli* (*E. coli*) and multidrug-resistant *Proteus mirabilis* (*P. mirabilis*), both isolated from urine cultures of patients with urinary tract infections. Their *in vitro* antibacterial susceptibility was then evaluated. The strains were inoculated into Luria–Bertani (LB) medium and incubated for 12 h at 37 °C to resuscitate. we determined the minimum inhibitory concentration (MIC) of Cu-ZIF8-Ru against the most common clinical MDR *E. coli* to identify optimal antibacterial incubation conditions using the Alamar Blue assay. To better mimic the urinary-tract microenvironment, four incubation settings were established: Control, H₂O₂, Laser, and H₂O₂ + Laser. Each was tested at pH 7.4 and pH 5.5, with H₂O₂ fixed at 200 µM. MDR *E. coli* was cultured in LB broth to the logarithmic phase and diluted to 1 × 10⁶ CFU/mL. Cu-ZIF8-Ru was prepared as a concentration series (0, 12.5, 25, 50, 75, 100, 150, and 200 µg/mL). Bacterial suspensions and the corresponding sample concentrations were co-incubated in 96-well plates for 5 h, after which the Laser groups were exposed to 660 nm irradiation at 1 W/cm² for 10 min. After a total of 12 h incubation, Alamar Blue (10% v/v) was added to each well and incubation continued for 4 h at constant temperature. Wells were photographed, and bacterial activity was judged by color change: pink/red indicates active growth; blue-violet indicates inhibition or death. Fluorescence was measured on a microplate reader (excitation 540 nm, emission 590 nm) to evaluate the effect of Cu-ZIF8-Ru on MDR *E. coli* viability and to confirm the optimal antibacterial incubation conditions.

**Antibacterial activity.** Under the incubation conditions optimized above, the antibacterial activity of Cu-ZIF8-Ru against MDR *E. coli* and MDR *P. mirabilis* was further verified. Groups included: Ru(II), Cu-ZIF8, Cu-ZIF8-Ru, Ru(II)+Laser, Cu-ZIF8+Laser, and Cu-ZIF8-Ru+Laser. MDR *E. coli* and MDR *P. mirabilis* were cultured in LB broth to the logarithmic phase and diluted to 1 × 10⁶ CFU/mL. Each group was prepared as a concentration series (0, 12.5, 25, 50, 75, 100, 150, 200 µg/mL, quantified by Cu-ZIF8-Ru concentration). Bacterial suspensions were mixed with the corresponding concentrations of Ru(II), Cu-ZIF8, or Cu-ZIF8-Ru in 96-well plates and incubated for 5 h at pH 5.5 in the presence of H₂O₂. The Laser groups were then irradiated at 660 nm (1 W/cm²) for 10 min. After a total of 12 h incubation, Alamar Blue (10% v/v) was added to each well and plates were further incubated for 4 h. Wells were photographed and bacterial activity was judged by color change, and fluorescence was measured on a microplate reader (excitation 540 nm, emission 590 nm) to evaluate the effects of each group on MDR *E. coli* and MDR *P. mirabilis*, and to confirm the optimal antibacterial incubation conditions.

**Bacterial plating assay.** MDR *E. coli* and MDR *P. mirabilis* were cultured in LB broth to the logarithmic phase and diluted to 1 × 10⁶ CFU/mL. Eight groups were established: Control, Ru(II), Cu-ZIF8, Cu-ZIF8-Ru, Laser, Ru(II)+Laser, Cu-ZIF8+Laser, and Cu-ZIF8-Ru+Laser. In 24-well plates, bacterial suspensions were mixed with Ru(II), Cu-ZIF8, or Cu-ZIF8-Ru at the same concentration of 50 µg/mL (quantified by Cu-ZIF8-Ru) and incubated for 5 h at pH 5.5 with H₂O₂. The Laser groups were then irradiated at 660 nm (1 W/cm²) for 10 min. After a total of 12 h incubation, the suspensions from all eight groups were diluted 10⁴-fold, and 40 µL of each was spread onto LB agar plates. Plates were incubated at 37 °C for 15 h. Colony numbers were quantified using ImageJ, and bacterial survival rates were calculated.

**Live/Dead bacterial assay.** MDR *E. coli* and MDR *P. mirabilis* were cultured in LB broth to the logarithmic phase and diluted to 1 × 10⁶ CFU/mL. Suspensions were incubated for 5 h under pH 5.5 in the presence of H₂O₂. Eight groups were established: Control, Ru(II), Cu-ZIF8, Cu-ZIF8-Ru, Laser, Ru(II)+Laser, Cu-ZIF8+Laser, and Cu-ZIF8-Ru+Laser. In 24-well plates, bacteria were mixed with Ru(II), Cu-ZIF8, or Cu-ZIF8-Ru at the same concentration of 50 µg/mL (dose referenced to Cu-ZIF8-Ru) and incubated for 5 h; Laser groups were then irradiated at 660 nm (1 W/cm²) for 10 min. After a total of 12 h incubation, SYTO-9 and PI were added, followed by a 20-min incubation at 37 °C. Fluorescence images of stained bacteria were acquired by fluorescence microscopy.

**Intracellular ROS assay in bacteria.** MDR *E. coli* and MDR *P. mirabilis* were cultured in LB broth to the logarithmic phase and diluted to 1 × 10⁶ CFU/mL. Suspensions were incubated for 5 h at pH 5.5 in the presence of H₂O₂. Eight groups were established: Control, Ru(II), Cu-ZIF8, Cu-ZIF8-Ru, Laser, Ru(II)+Laser, Cu-ZIF8+Laser, and Cu-ZIF8-Ru+Laser. In 24-well plates, bacteria were mixed with Ru(II), Cu-ZIF8, or Cu-ZIF8-Ru at the same concentration of 50 µg/mL (dose referenced to Cu-ZIF8-Ru) and incubated for 5 h; Laser groups were then irradiated at 660 nm (1 W/cm²) for 10 min. After a further 2 h, bacteria were treated with the ROS probe DCFH-DA and incubated for 20 min at 37 °C. Samples were washed three times with PBS, and fluorescence images of stained bacteria were acquired by fluorescence microscopy.

**Bacterial morphology.** MDR *E. coli* and MDR *P. mirabilis* were cultured in LB broth to the logarithmic phase and diluted to 1 × 10⁶ CFU/mL. Suspensions were incubated for 5 h at pH 5.5 in the presence of H₂O₂. Eight groups were established: Control, Ru(II), Cu-ZIF8, Cu-ZIF8-Ru, Laser, Ru(II)+Laser, Cu-ZIF8+Laser, and Cu-ZIF8-Ru+Laser. In 24-well plates, bacteria were mixed with Ru(II), Cu-ZIF8, or Cu-ZIF8-Ru at the same concentration of 50 µg/mL (dose referenced to Cu-ZIF8-Ru) and incubated for 5 h; Laser groups were then irradiated at 660 nm (1 W/cm²) for 10 min. After 7 h of incubation, treated suspensions from each group were collected, washed three times with PBS, and immediately fixed with 2.5% glutaraldehyde for 30 min. Samples were then dehydrated through a graded ethanol series and examined by SEM to assess morphological changes.

**antibiofilm properties.** (1) Biofilm disruption assay. MDR *E. coli* and MDR *P. mirabilis* were grown in LB broth to the logarithmic phase. A total of 100 µL of bacterial suspension (1 × 10⁸ CFU/mL) and 100 µL of LB medium were added to each well of a 96-well plate and incubated at 37 °C for 48 h to form mature biofilms. Groups were: Control, Ru(II), Cu-ZIF8, Cu-ZIF8-Ru, Laser, Ru(II)+Laser, Cu-ZIF8+Laser, and Cu-ZIF8-Ru+Laser. After biofilm formation, wells were washed three times with PBS to remove planktonic bacteria. Then 50 µg/mL (dose referenced to Cu-ZIF8-Ru) of Ru(II), Cu-ZIF8, or Cu-ZIF8-Ru was added and incubated for 5 h; Laser groups were irradiated at 660 nm (1 W/cm²) for 10 min. After a further 7 h, 10 µL of 0.1% crystal violet was added per well and incubated for 30 min at constant temperature. Wells were photographed, air-dried, the residual crystal violet bound to biofilms was solubilized with methanol, and absorbance was measured at 570 nm (OD570) on a microplate reader. (2) Biofilm inhibition assay. Log-phase MDR *E. coli* and MDR *P. mirabilis* were prepared as above, and 100 µL of bacterial suspension (1 × 10⁸ CFU/mL) plus 100 µL LB were added to 96-well plates. Ru(II), Cu-ZIF8, or Cu-ZIF8-Ru (each 50 µg/mL, quantified by Cu-ZIF8-Ru) was added; after 5 h incubation, Laser groups were irradiated at 660 nm (1 W/cm²) for 10 min. After an additional 7 h, 10 µL of 0.1% crystal violet was added and incubated 30 min. Wells were photographed, dried, stained dye was dissolved with methanol, and OD570 was recorded to quantify biofilm formation.

**Antibacterial Mechanism**

**Surface adhesion of Cu-ZIF8-Ru.** MDR *E. coli* and MDR *P. mirabilis* were cultured in LB broth to the logarithmic phase and diluted to 1 × 10⁶ CFU/mL. Suspensions were incubated for 5 h at pH 5.5 in the presence of H₂O₂. Eight groups were set up: Control, Ru(II), Cu-ZIF8, Cu-ZIF8-Ru, Laser, Ru(II)+Laser, Cu-ZIF8+Laser, and Cu-ZIF8-Ru+Laser. In 24-well plates, bacteria were mixed with Ru(II), Cu-ZIF8, or Cu-ZIF8-Ru at the same concentration of 50 µg/mL (dose referenced to Cu-ZIF8-Ru) and incubated for 5 h; Laser groups were then irradiated at 660 nm (1 W/cm²) for 10 min. After 7 h of incubation, treated suspensions from each group were collected, immediately fixed with 2.5% glutaraldehyde for 30 min, dehydrated through a graded ethanol series, and examined by TEM to observe the morphology of Cu-ZIF8-Ru on bacterial surfaces.

**Ultrastructural changes in bacteria.** MDR *E. coli* and MDR *P. mirabilis* were cultured in LB broth to the logarithmic phase and diluted to 1 × 10⁶ CFU/mL, then incubated for 5 h at pH 5.5 in the presence of H₂O₂. Eight groups were established: Control, Ru(II), Cu-ZIF8, Cu-ZIF8-Ru, Laser, Ru(II)+Laser, Cu-ZIF8+Laser, and Cu-ZIF8-Ru+Laser. In 24-well plates, bacteria were mixed with Ru(II), Cu-ZIF8, or Cu-ZIF8-Ru at 50 µg/mL (dose referenced to Cu-ZIF8-Ru) and incubated for 5 h; laser groups were then irradiated at 660 nm (1 W/cm²) for 10 min. After 7 h of incubation, suspensions from each group were collected, washed three times with PBS, immediately fixed with 2.5% glutaraldehyde for 30 min, and dehydrated through a graded ethanol series. The pellets were collected for cryo-sectioning, and the ultrastructural changes of bacteria after Cu-ZIF8-Ru treatment were examined by TEM.

**Intrabacterial elemental distribution.** MDR *E. coli* and MDR *P. mirabilis* were cultured in LB broth to the logarithmic phase and diluted to 1 × 10⁶ CFU/mL, then incubated for 5 h at pH 5.5 in the presence of H₂O₂. Eight groups were established: Control, Ru(II), Cu-ZIF8, Cu-ZIF8-Ru, Laser, Ru(II)+Laser, Cu-ZIF8+Laser, and Cu-ZIF8-Ru+Laser. In 24-well plates, bacteria were mixed with Ru(II), Cu-ZIF8, or Cu-ZIF8-Ru at the same concentration of 50 µg/mL (dose referenced to Cu-ZIF8-Ru) and incubated for 5 h; the Laser groups were then irradiated at 660 nm (1 W/cm²) for 10 min. After 7 h of incubation, suspensions from each group were collected, washed three times with PBS, immediately fixed with 2.5% glutaraldehyde for 30 min, and dehydrated through a graded ethanol series. The bacterial samples were then dropped onto copper grids, air-dried at room temperature, and directly analyzed by TEM-EDS.

**Bacterial membrane permeability.** MDR *E. coli* and MDR *P. mirabilis* were cultured in LB broth to the logarithmic phase and diluted to 1 × 10⁶ CFU/mL, then incubated for 5 h at pH 5.5 in the presence of H₂O₂. Eight groups were set up: Control, Ru(II), Cu-ZIF8, Cu-ZIF8-Ru, Laser, Ru(II)+Laser, Cu-ZIF8+Laser, and Cu-ZIF8-Ru+Laser. In 24-well plates, bacteria were mixed with Ru(II), Cu-ZIF8, or Cu-ZIF8-Ru at the same concentration of 50 µg/mL (dose referenced to Cu-ZIF8-Ru) and incubated for 5 h; Laser groups were then irradiated at 660 nm (1 W/cm²) for 10 min. After 7 h of incubation, treated suspensions were collected, PI probe was added, and samples were incubated for 20 min at 37 °C. Fluorescence was measured on a microplate reader (excitation 493 nm, emission 636 nm) to evaluate membrane-permeability changes in MDR *E. coli* and MDR *P. mirabilis* across groups.

**Bacterial membrane potential.** MDR *E. coli* and MDR *P. mirabilis* were cultured in LB broth to the logarithmic phase and diluted to 1 × 10⁶ CFU/mL, then incubated for 5 h at pH 5.5 in the presence of H₂O₂. Eight groups were established: Control, Ru(II), Cu-ZIF8, Cu-ZIF8-Ru, Laser, Ru(II)+Laser, Cu-ZIF8+Laser, and Cu-ZIF8-Ru+Laser. In 24-well plates, bacteria were mixed with Ru(II), Cu-ZIF8, or Cu-ZIF8-Ru at the same concentration of 50 µg/mL (dose referenced to Cu-ZIF8-Ru) and incubated for 5 h; Laser groups were then irradiated at 660 nm (1 W/cm²) for 10 min. After 7 h of incubation, the suspensions were collected, the DiSC₃(5) probe was added, and samples were incubated for 20 min at 37 °C. Fluorescence was measured on a microplate reader (excitation 622 nm, emission 670 nm) to assess membrane potential depolarization across treatment groups.

**Protein leakage.** MDR *E. coli* and MDR *P. mirabilis* were cultured in LB broth to the logarithmic phase and diluted to 1 × 10⁶ CFU/mL, then incubated for 5 h at pH 5.5 in the presence of H₂O₂. Groups were Triton X-100, Control, Ru(II), Cu-ZIF8, Cu-ZIF8-Ru, Laser, Ru(II)+Laser, Cu-ZIF8+Laser, and Cu-ZIF8-Ru+Laser. In 24-well plates, bacteria were mixed with Ru(II), Cu-ZIF8, or Cu-ZIF8-Ru at the same concentration of 50 µg/mL (dose referenced to Cu-ZIF8-Ru) and incubated for 5 h; Laser groups were then irradiated at 660 nm (1 W/cm²) for 10 min. After a further 7 h, suspensions were collected and centrifuged to pellet the bacteria. The pellets were sonicated to disrupt cell envelopes, followed by centrifugation to obtain supernatants. According to the BCA kit instructions, BCA working solution was added to each supernatant and the mixtures were incubated at 37 °C for 20 min. Absorbance at 562 nm was measured on a microplate reader to quantify the amount of leaked protein.

**DNA leakage.** MDR *E. coli* and MDR *P. mirabilis* were cultured in LB broth to the logarithmic phase and diluted to 1 × 10⁶ CFU/mL, then incubated for 5 h at pH 5.5 in the presence of H₂O₂. Groups included Triton X-100, Control, Ru(II), Cu-ZIF8, Cu-ZIF8-Ru, Laser, Ru(II)+Laser, Cu-ZIF8+Laser, and Cu-ZIF8-Ru+Laser. In 24-well plates, bacteria were mixed with Ru(II), Cu-ZIF8, or Cu-ZIF8-Ru at the same concentration of 50 µg/mL (dose referenced to Cu-ZIF8-Ru) and incubated for 5 h; Laser groups were then irradiated at 660 nm (1 W/cm²) for 10 min. After a further 7 h, suspensions were collected and centrifuged to pellet bacteria. Pellets were sonicated to disrupt the cell envelope and then centrifuged to obtain supernatants. DNA released into the supernatant was quantified by measuring absorbance at 260 nm on a microplate reader.

**Bacterial ATP content.** MDR *E. coli* and MDR *P. mirabilis* were cultured in LB broth to the logarithmic phase and diluted to 1 × 10⁶ CFU/mL, then incubated for 5 h at pH 5.5 in the presence of H₂O₂. Eight groups were established: Control, Ru(II), Cu-ZIF8, Cu-ZIF8-Ru, Laser, Ru(II)+Laser, Cu-ZIF8+Laser, and Cu-ZIF8-Ru+Laser. In 24-well plates, bacteria were mixed with Ru(II), Cu-ZIF8, or Cu-ZIF8-Ru at the same concentration of 50 µg/mL (dose referenced to Cu-ZIF8-Ru) and incubated for 5 h; Laser groups were then irradiated at 660 nm (1 W/cm²) for 10 min. After 7 h of incubation, suspensions from each group were collected and subjected to repeated freeze–thaw cycles at −80 °C to disrupt the membranes, followed by centrifugation to obtain supernatants. An ATP assay kit was added to each supernatant, and the mixtures were incubated at 37 °C for 20 min. Fluorescence intensity was measured on a microplate reader to quantify ATP released.

**Bacterial lipid peroxidation.** MDR *E. coli* was cultured in LB broth to the logarithmic phase and diluted to 1 × 10⁶ CFU/mL, then incubated for 5 h at pH 5.5 in the presence of H₂O₂. Four groups were set up: Control, Cu²⁺, Cu-ZIF8-Ru, and Cu-ZIF8-Ru+Laser. In 24-well plates, bacterial suspensions were mixed with Cu²⁺, Cu-ZIF8-Ru, or Cu-ZIF8-Ru+Laser at the same concentration of 15 µg/mL (dose quantified by Cu²⁺) and incubated for 5 h; the Laser group was then irradiated at 660 nm (1 W/cm²) for 10 min. After another 5 h, suspensions were collected and subjected to repeated freeze–thaw cycles at −80 °C to disrupt membranes, followed by centrifugation to obtain supernatants. An MDA assay kit was applied to each supernatant, and absorbance at 532 nm was measured on a microplate reader to quantify MDA content and evaluate lipid peroxidation levels.

**Bacterial pyruvate dehydrogenase (PDH) activity.** MDR *E. coli* was cultured in LB broth to the logarithmic phase and diluted to 1 × 10⁶ CFU/mL, then incubated for 5 h at pH 5.5 in the presence of H₂O₂. Four groups were set up: Control, Cu²⁺, Cu-ZIF8-Ru, and Cu-ZIF8-Ru + Laser. In 24-well plates, bacterial suspensions were mixed with Cu²⁺, Cu-ZIF8-Ru, or Cu-ZIF8-Ru + Laser at the same concentration of 15 µg/mL (dose quantified by Cu²⁺) and incubated for 5 h; the Laser group was then irradiated at 660 nm (1 W/cm²) for 10 min.After another 5 h, suspensions were collected and subjected to repeated freeze–thaw cycles at −80 °C to disrupt membranes, followed by centrifugation to obtain supernatants. A PDH activity assay kit was applied to each supernatant, and absorbance at 605 nm was measured on a microplate reader to quantify PDH levels.

**Bacterial transcriptome analysis.** MDR *E. coli* was cultured in LB broth to the logarithmic phase, diluted to 1 × 10⁶ CFU/mL, and incubated for 5 h at pH 5.5 in the presence of H₂O₂. The experimental groups were Control, Cu²⁺, Cu-ZIF8-Ru, and Cu-ZIF8-Ru + Laser. In 24-well plates, bacterial suspensions were mixed with the corresponding agents at an equivalent dose of 15 µg/mL (quantified by Cu²⁺) and incubated for 5 h; cells in the Laser group were then irradiated at 660 nm (1 W/cm²) for 10 min. After a further 5 h, treated suspensions were collected and snap-frozen in liquid nitrogen, then submitted to Shanghai Majorbio for prokaryotic transcriptome sequencing, including RNA extraction, sequencing, and initial analysis. RNA-seq was performed on the Illumina NovaSeq X Plus platform. Bioinformatic analysis was conducted on data generated by the Illumina platform. In addition to the default RSEM (<http://deweylab.github.io/RSEM/>), expression quantification was performed using Kallisto (<https://pachterlab.github.io/kallisto/>) and Salmon (<https://combine-lab.github.io/salmon/>). Differentially expressed genes (DEGs) were defined as those with |log₂FoldChange| > 1 and adjusted *p* value (padj) < 0.05. Functional annotation and enrichment clustering were carried out using the Gene Ontology (GO) and Kyoto Encyclopedia of Genes and Genomes (KEGG) databases.

***In vivo* antibacterial efficacy.** All animal experiments were approved by the Institutional Animal Care and Use Committee of the Second Affiliated Hospital of Guangzhou Medical University (approval No. A2020-036, experimental animal use license: SYXK 2022-0298). Healthy female C57BL/6J mice (6-8 weeks old) were supplied by Guangdong Experimental Animal Technology. After a 7 days acclimatization, the mice underwent different experiments. Animals were maintained under SPF conditions. All rats were housed in high-pressure micro-isolator cages and provided sterile drinking water with food ad libitum. During the experiments, rats were kept at room temperature with 55% humidity under a 12-h light/dark cycle. One week before the start of experiments, each rat acclimated to the animal room environment.

**Cu-ZIF8-Ru@Gel nanozyme hydrogel sustained-release assay.** (1) 50 µL of Cu-ZIF8-Ru@Gel were allowed to gel at 37 °C into a flat spheroid, weighed, and recorded. The gel was then placed in 2 mL PBS or artificial urine and gently stirred at 200 rpm. PBS or artificial urine was replaced every 4 h. Every 6 h, the supernatant was removed, and the gel was weighed and recorded. The dissolution process of Cu-ZIF8-Ru@Gel in PBS and artificial urine was observed and documented. (2) 50 µL of Cu-ZIF8-Ru@Gel were gelled at 37 °C and placed in 2 mL artificial urine with gentle stirring at 200 rpm. Every 6 h, the supernatant was collected, and absorbance at 445 nm was measured on a microplate spectrophotometer and recorded, continuing until the Cu-ZIF8-Ru@Gel was essentially dissolved.

**Intravesical sustained-release test of ICG-labeled Cu-ZIF8-Ru@Gel.** Six healthy female C57BL/6 mice (6-8 weeks old) were fasted from water for 4 h. Two groups were set up: ICG-labeled Cu-ZIF8-Ru and ICG-labeled Cu-ZIF8-Ru@Gel. A 50 µL dose of the respective formulation was instilled into the bladder via catheter. Drinking water was restored 2 h after instillation. Whole-body fluorescence distributions were recorded at 0.5, 1.5, 2, 3, 4, 6, 8, and 10 h using an IVIS Lumina III *in vivo* imaging system.

**Acute Urinary Tract Infections Model:** MDR *E. coli* was cultured in LB liquid medium to the logarithmic growth phase, and the bacterial suspension concentration was adjusted to approximately 1×10⁸ CFU/mL using a dilution method. Before the experiment, female C57BL/6 mice were deprived of water for 4 hours, anesthetized, and subjected to abdominal hair removal. A 1 mL syringe fitted with a plastic catheter was inserted into the bladder via the urethra to a depth of about 1.5-2 cm. The bladder was then flushed with 0.1–0.2 mL of saline to remove residual urine. Subsequently, 50 µL of the bacterial suspension (1×10⁸ CFU/mL) was injected into the bladder, allowed to dwell for 30 seconds, and the catheter was slowly withdrawn. Finally, the syringe needle was removed, and the mice were placed on a heating pad with close monitoring to ensure survival. After recovery, the mice were returned to their cages, and water access was restored 2 hours later. Successful establishment of the acute UTIs model was confirmed by H&E staining 6 hours after bacterial instillation. After establishing the acute UTI model, the mice were divided into 6 groups (n=6 per group): (1) Uninfected, (2) Control, (3) Cu-ZIF8, (4) Cu-ZIF8-Ru, (5) Cu-ZIF8-Ru@Gel, and (6) Cu-ZIF8-Ru@Gel + Light. Six hours after bacterial instillation, drug treatment was administered via intravesical instillation of 50 µL of Cu-ZIF8, Cu-ZIF8-Ru, and Cu-ZIF8-Ru@Gel at 50 µg/mL (dose quantified by Cu-ZIF8-Ru). One hour later, mice in the laser group were exposed to 660 nm laser irradiation at a power density of 1 W/cm² for 10 minutes over the bladder region. Finally, 18 hours after bacterial instillation, the mice were euthanized under anesthesia by cervical dislocation. Bladder, kidney, and other major organs were collected for analysis. The harvested bladders were photographed, then bisected crosswise: one half was processed for histological sectioning and staining, and the other half was homogenized in LB liquid medium and incubated at 37°C for 8 hours for bacterial culture. After incubation, the culture broth from each group was plated and further incubated at 37°C for 15 hours. Bacterial colony counts were determined using ImageJ, and the survival rate of bacteria in the bladder was calculated.

**Recurrent Urinary Tract Infections Model:** MDR *E. coli* was cultured in LB liquid medium to the logarithmic growth phase, and the bacterial suspension concentration was adjusted to approximately 1×10⁶ CFU/mL using a dilution method. Before the experiment, female C57BL/6 mice were deprived of water for 4 hours, anesthetized, and subjected to abdominal hair removal. A 1 mL syringe fitted with a plastic catheter was inserted into the bladder via the urethra to a depth of about 1.5-2 cm. The bladder was then flushed with 0.1–0.2 mL of saline to remove residual urine. Subsequently, 50 µL of the bacterial suspension (1×10⁶ CFU/mL) was instilled into the bladder, retained for 30 seconds, after which the catheter was slowly withdrawn. Finally, the syringe needle was removed, and the mice were placed on a heating pad and closely monitored to ensure survival. After recovery, the mice were returned to their cages, and water access was restored 2 hours later. This infection procedure was repeated every 4 days for a total of 3 cycles to establish the recurrent UTI model, which was subsequently confirmed by H&E staining. Following model establishment, the mice were divided into 6 groups (n=6 per group): (1) Uninfected, (2) Control, (3) Cu-ZIF8, (4) Cu-ZIF8-Ru, (5) Cu-ZIF8-Ru@Gel, and (6) Cu-ZIF8-Ru@Gel + Light. The mice received intravesical instillation of 50 µL of Cu-ZIF8, Cu-ZIF8-Ru, and Cu-ZIF8-Ru@Gel at 50 µg/mL (dose quantified by Cu-ZIF8-Ru). One hour later, mice in the laser group were exposed to 660 nm laser irradiation at a power density of 1 W/cm² over the bladder region for 10 minutes. The treatment was administered three times in total. The blood samples were collected from all mice after the treatment. The mice were euthanized by cervical dislocation under anesthesia, and the major organs were harvested. The collected bladder and kidney tissues were photographed. The bladder and kidneys organ was bisected crosswise: one half was processed for histological sectioning and staining, and the other half was homogenized in LB liquid medium and incubated at 37°C for 8 hours for bacterial culture. Following incubation, the culture broth from each group was plated and further incubated at 37°C for 15 hours. Bacterial colony counts were determined using ImageJ, and the survival rate of bacteria in the bladder and kidney was calculated accordingly.

**Statistical analysis**

All experiments were performed with at least three independent replicates (n ≥ 3). Data are presented as mean ± standard deviation (SD). Statistical comparisons between two groups were conducted using an unpaired two-tailed Student’s t-test. For comparisons across multiple groups, one-way analysis of variance (ANOVA) was applied. A P value of less than 0.05 was considered statistically significant (*P < 0.05, **P < 0.01, ***P < 0.001, ****P < 0.0001). All analyses were performed using GraphPad Prism software (version 9).

**Figure S1.** TEM images of Cu-ZIF8 with different copper contents.

**Figure S2.** TEM images of Cu-ZIF8 **(A)** and Cu-ZIF8-Ru **(B)**.

**Figure S3.** Particle size distribution of Cu-ZIF8 and Cu-ZIF8-Ru.

**Figure S4.** EDS spectrum confirming the presence of C, N, O, Zn, Cu, and Ru elements in Cu-ZIF8-Ru.

**Figure S5.** XRD peak shifts of Cu-ZIF8 and Cu-ZIF8-Ru at the 011 **(A)** and 202 **(B)** plane**.**

**Figure S6.** UV-visible absorption of Ru(Ⅱ) and Cu-ZIF8-Ru diluted in concentration gradient at a wavelength of 445 nm

**Figure S7.** FTIR spectra of Metal-N stretching vibration with a bule shift from 421.8 cm⁻¹ (Cu-ZIF8) to 419.4 cm⁻¹ (Cu-ZIF8-Ru)

**Figure S8.** High-resolution XPS spectra of Zn 2p, displaying Zn 2p₃/₂ and Zn 2p₁/₂ peaks

**Figure S9. (A)** Ru K-edge derivative spectra of Cu-ZIF8-Ru, Ru foil, and RuO₂, revealing an oxidation state of Ru in Cu-ZIF8-Ru close to +2. **(B)** Cu K-edge derivative spectra of Cu-ZIF8-Ru, Cu-ZIF8, Cu foil, and CuO, indicating a similar +2 oxidation state for Cu in both Cu-ZIF8 and Cu-ZIF8-Ru.

**Figure S10. (A)** WT images of RuO_2_ of the Ru K-edge. **(B)** WT images of CuO of the Cu K-edge.

**Figure S11.** Time-dependent GSH depletion of Cu-ZIF8 at 50 μg mL^−1^ using the DTNB probe

**Figure S12. Charge density difference analysis. (A)** Charge density distribution of Cu-ZIF8. **(B)** Charge density distribution of Cu-ZIF8-Ru.

**Figure S13.** DFT-optimized intermediate structures and reaction pathways of O₂ reduction **(A)**, H₂O₂ decomposition **(B)**, GSH oxidation **(C)** in Cu-ZIF8.

**Figure S14.** Morphological changes of Cu-ZIF8-Ru of progressive structural degradation under acidic environments at pH 7.4, pH 6.5, and pH 5.5.

**Figure S15.** The UV-Vis absorption of Cu-ZIF8-Ru at 450 nm was detected with the TMB probe following incubation in acidic solution for different time periods.

**Figure S16.** **Cytotoxicity assessment of Cu-ZIF8-Ru *in vitro*.** (**A**, **B**) Viability of SV-HUC-1 and HUVEC cells after treatment with increasing concentrations of Cu-ZIF8-Ru (with copper contents of 10%, 20%, and 30%). (**C**, **D**) Viability of SV-HUC-1 cells quantified after treatment with Ru(II), Cu-ZIF8, or Cu-ZIF8-Ru, without or with laser irradiation. Data are presented as the mean ± SD from three independent experiments, each with n = 4 replicates. Error bars represent SD.

**Figure S17.** Hemolysis assay of Cu-ZIF8-Ru. Values represent the mean ± SD from three independent experiments (n = 4). Error bars indicate SD.

**Figure S18. (A, B)** The antibacterial activity of *E. coli* and *P. mirabilis* cultured under different conditions (pH 7.4, pH 5.5, pH 7.4 + H₂O₂, pH 5.5 + H₂O₂). **(C)** Quantitative bacterial viability of *E. coli* and *P. mirabilis* under the same conditions. Values represent the mean ± SD from three independent experiments (n = 4). Error bars indicate SD.

**Figure S19. MDR *E. coli* viability under different conditions.** **(A)** Alamar Blue assay showing bacterial viability of cultures treated with different concentrations (0-200 μg mL⁻¹) under pH 7.4 and pH 5.5 with/without H₂O₂ and laser irradiation (Pink/red indicates active bacterial growth, while blue/purple indicates bacterial activity is inhibited or dead). **(B, C)** Quantitative viability analysis at pH 7.4 **(B)** and pH 5.5 **(C)**, demonstrating concentration-dependent antibacterial effects enhanced by H₂O₂ and laser. Values represent the mean ± SD from three independent experiments (n = 4). Error bars indicate SD.

**Figure S20.** Quantitative viability analysis of *E. coli* viability under different conditions. Values represent the mean ± SD from three independent experiments (n = 4). Error bars indicate SD.

**Figure S21.** Quantitative viability analysis of *P. mirabilis* viability under different conditions. Values represent the mean ± SD from three independent experiments (n = 4). Error bars indicate SD.

**Figure S22. ROS generation and antibacterial activity of different treatments. (A)** ROS intensity in MDR *E. coli* under various treatments. **(B)** ROS intensity in MDR *P. mirabilis* under various treatments. **(C)** Colony formation assay showing relative bacterial viability of MDR *E. coli*. **(D)** Colony formation assay showing relative bacterial viability of MDR *P. mirabilis*. Values represent the mean ± SD from three independent experiments (n = 3). Error bars indicate SD.

**Figure S23. Biofilm inhibition and destruction of MDR *E. coli* under different treatments. (A)** Crystal violet staining images of biofilm inhibition without and with laser irradiation. **(B)** Quantitative analysis of biofilm inhibition rate. **(C)** Crystal violet staining images of biofilm destruction without and with laser irradiation. **(D)** Quantitative analysis of biofilm destruction rate. Values represent the mean ± SD from three independent experiments (n = 3). Error bars indicate SD.

**Figure S24. Biofilm inhibition and destruction of MDR *P. mirabilis* under different treatments. (A)** Crystal violet staining images of biofilm inhibition without and with laser irradiation. **(B)** Quantitative analysis of biofilm inhibition rate. **(C)** Crystal violet staining images of biofilm destruction without and with laser irradiation. **(D)** Quantitative analysis of biofilm destruction rate. Values represent the mean ± SD from three independent experiments (n = 3). Error bars indicate SD.

**Figure S25. PI fluorescence assay of bacterial membrane damage. (A)** PI fluorescence intensity of E. coli after different treatments with and without laser irradiation. **(B)** PI fluorescence intensity of P. mirabilis after different treatments with and without laser irradiation. Values represent the mean ± SD from three independent experiments (n = 3). Error bars indicate SD.

**Figure S26.** Principal component analysis (PCA) score plot analysis of bacterial transcriptomic profiles**.**

**Figure S27. Volcano plots of differentially expressed genes (DEGs) under various treatments.** **(A)** DEGs between Cu²⁺ and Control. **(B)** DEGs between Cu-ZIF8-Ru and Control. **(C)** DEGs between Cu-ZIF8-Ru+Laser and Control. **(D)** DEGs between Cu-ZIF8-Ru+Laser and Cu-ZIF8-Ru.

**Figure S28. GO enrichment analysis of differentially expressed genes (DEGs) under various treatments.** (A) GO enrichment of upregulated DEGs in Cu-ZIF8-Ru vs Cu²⁺. **(B)** GO enrichment of downregulated DEGs in Cu-ZIF8-Ru vs Cu²⁺. **(C)** GO enrichment of upregulated DEGs in Cu-ZIF8-Ru+Laser vs Cu²⁺. **(D)** GO enrichment of downregulated DEGs in Cu-ZIF8-Ru+Laser vs Cu²⁺.

**Figure S29. KEGG pathway enrichment analysis of differentially expressed genes (DEGs) under various treatments.** **(A)** KEGG enrichment of DEGs in Cu-ZIF8-Ru vs Cu²⁺. **(B)** KEGG enrichment of upregulated DEGs in Cu-ZIF8-Ru vs Cu^2+^. **(C)** KEGG enrichment of DEGs in Cu-ZIF8-Ru+Laser vs Cu^2+^. **(D)** KEGG enrichment of upregulated DEGs in Cu-ZIF8-Ru+Laser vs Cu^2+^.

**Figure S30.** (A, B) H&E of bladder tissues in the acute urinary tract infection (UTIs) pre-experiment.

**Figure S31. Rheological characterization of PLGA-PEG-PLGA and Cu-ZIF8-Ru@Gel. (A)** Storage modulus (G′) and loss modulus (G″) of PLGA-PEG-PLGA as a function of temperature. **(B)** Viscosity (η) of PLGA-PEG-PLGA. **(C)** Viscosity (η) of Cu-ZIF8-Ru@Gel.

**Figure S32. Degradation and drug release behavior of Cu-ZIF8-Ru@Gel.** **(A)** Photographs showing the appearance of Cu-ZIF8-Ru@Gel in PBS and artificial urine at 0 h and 30 h. **(B)** Quantitative degradation profiles of Cu-ZIF8-Ru@Gel in PBS and artificial urine within 30 h. **(C)** Cumulative release of Cu-ZIF8-Ru from Cu-ZIF8-Ru@Gel over time in artificial urine.

**Figure S33.** *In vivo* bioluminescence imaging of mice at different time points after intravesical instillation of ICG-labeled Cu-ZIF8-Ru.

**Figure S34.** H&E staining of kidney tissues from different groups in acute urinary tract infection model.

**Figure S35.** H&E staining of major organs from different groups in acute urinary tract infection model. Histological images of the heart, liver, lung and spleen collected from (1) Un-infection, (2) Control, (3) Cu-ZIF8, (4) Cu-ZIF8-Ru, (5) Cu-ZIF8-Ru@Gel, and (6) Cu-ZIF8-Ru@Gel+Laser groups.

**Figure S36.** **(A, B)** H&E of bladder tissues in the repeated urinary tract infection (UTIs) pre-experiment.

**Figure S37.** Histological and immunohistochemical analysis of kidney tissues in repeated urinary tract infection model. Images of H&E staining and immunohistochemical staining for CD68, IL-6, and TNF-α in kidney sections from (1) Un-infection, (2) Control, (3) Cu-ZIF8, (4) Cu-ZIF8-Ru, (5) Cu-ZIF8-Ru@Gel, and (6) Cu-ZIF8-Ru@Gel+Laser groups.

**Figure S38.** Hematological analysis of mice from different groups in repeated urinary tract infection model. Blood routine parameters including **(A)** red blood cell count (RBC), **(B)** hemoglobin (HGB), and **(C)** platelet count (PLT).

**Figure S39.** H&E staining of major organs from different groups in repeated urinary tract infection model. Histological images of the heart, liver, lung and spleen collected from (1) Un-infection, (2) Control, (3) Cu-ZIF8, (4) Cu-ZIF8-Ru, (5) Cu-ZIF8-Ru@Gel, and (6) Cu-ZIF8-Ru@Gel+Laser groups.

**Table S1.** Quantitative EXAFS fitting results of Ru K-edge for Ru foil and Cu-ZIF8-Ru.

**Table S2.** Quantitative EXAFS fitting results of Cu K-edge for Cu foil, Cu-ZIF8, and Cu-ZIF8-Ru.

**Table S3.** Antimicrobial susceptibility profiles of ESBL-positive E. coli and P. mirabilis isolated from urine samples**.**

**References**

[1]	G. Li, S. Wu, J. Liu, et al., Narrow Bandgap Schottky Heterojunction Sonosensitizer with High Electron–Hole Separation Boosted Sonodynamic Therapy in Bladder Cancer. *Advanced Materials* **2024**, *36* (26), 2401252, <https://doi.org/https://doi.org/10.1002/adma.202401252>.

[2]	B. Ravel, M. Newville, ATHENA, ARTEMIS, HEPHAESTUS: data analysis for X-ray absorption spectroscopy using IFEFFIT. *Journal of Synchrotron Radiation* **2005**, *12* (4), 537, <https://doi.org/doi:10.1107/S0909049505012719>.

[3]	M. Newville, IFEFFIT : interactive XAFS analysis and FEFF fitting. *Journal of Synchrotron Radiation* **2001**, *8* (2), 322, <https://doi.org/doi:10.1107/S0909049500016964>.

[4]	G. Kresse, J. Furthmüller, Efficiency of ab-initio total energy calculations for metals and semiconductors using a plane-wave basis set. *Computational Materials Science* **1996**, *6* (1), 15, <https://doi.org/https://doi.org/10.1016/0927-0256(96)00008-0>.

[5]	Efficient iterative schemes for ab initio total-energy calculations using a plane-wave basis set. *Physical Review B* **1996**, *54* (16), 11169, <https://doi.org/10.1103/PhysRevB.54.11169>.

[6]	J. P. Perdew, K. Burke, M. Ernzerhof, Generalized Gradient Approximation Made Simple. *Physical Review Letters* **1996**, *77* (18), 3865, <https://doi.org/10.1103/PhysRevLett.77.3865>.

[7]	Blochl, Projector augmented-wave method. *Physical review. B, Condensed matter* **1994**, *50* (24), 17953, <https://doi.org/10.1103/PhysRevB.50.17953>.

[8]	R. P. Muller, A. E. Mattsson, C. L. Janssen, Calculation of Chemical Reaction Energies Using the AM05 Density Functional. *JOURNAL OF COMPUTATIONAL CHEMISTRY* **2010**, *31* (9), 1860, <https://doi.org/10.1002/jcc.21472>.
